# Tumor-suppressive miR-4732-3p is sorted into fucosylated exosome by hnRNPK to avoid the inhibition of lung cancer progression

**DOI:** 10.1186/s13046-024-03048-1

**Published:** 2024-04-23

**Authors:** Wanzhen Zhuang, Chengxiu Liu, Yilin Hong, Yue Zheng, Minjian Huang, Haijun Tang, Lilan Zhao, Zhixin Huang, Mingshu Tu, Lili Yu, Jianlin Chen, Yi Zhang, Xiongfeng Chen, Fan Lin, Qi Gao, Chundong Yu, Yi Huang

**Affiliations:** 1 Shengli Clinical Medical College, Fujian Medical University, Fuzhou, 350001 China; 2 Department of Clinical Laboratory, Fujian Provincial Hospital, Fuzhou, 350001 China; 3 State Key Laboratory of Cellular Stress Biology, Innovation Center for Cell Signaling Network, School of Life Sciences, Xiamen University, Xiamen, 361102 China; 4 Institute of Future Technology, Beijing Hotgen Biotech Co., Ltd, Beijing, 102600 China; 5 Center for Experimental Research in Clinical Medicine, Fujian Provincial Hospital, Fuzhou, 350001 China; 6 Department of Thoracic Surgery, Fujian Provincial Hospital, Fuzhou, 350001 China; 7 Integrated Chinese and Western Medicine College, Fujian University of Traditional Chinese Medicine, Fuzhou, 350108 China; 8 Department of Scientific Research, Fujian Provincial Hospital, Fuzhou, 350001 China; 9 Department of Geriatric Medicine, Fujian Provincial Hospital, Fuzhou, 350001 China; 10 Fujian Provincial Center for Geriatrics, Fujian Provincial Hospital, Fuzhou, 350001 China; 11 Central Laboratory, Fujian Provincial Hospital, Fuzhou, 350001 China; 12 Fujian Provincial Key Laboratory of Critical Care Medicine, Fujian Provincial Key Laboratory of Cardiovascular Disease, Fuzhou, 350001 China

**Keywords:** miR-4732-3p, Fucosylated exosome, hnRNPK, Exosome escape, MFSD12

## Abstract

**Background:**

Aberrant fucosylation observed in cancer cells contributes to an augmented release of fucosylated exosomes into the bloodstream, where miRNAs including miR-4732-3p hold promise as potential tumor biomarkers in our pilot study. However, the mechanisms underlying the sorting of miR-4732-3p into fucosylated exosomes during lung cancer progression remain poorly understood.

**Methods:**

A fucose-captured strategy based on lentil lectin-magnetic beads was utilized to isolate fucosylated exosomes and evaluate the efficiency for capturing tumor-derived exosomes using nanoparticle tracking analysis (NTA). Fluorescence in situ hybridization (FISH) and qRT-PCR were performed to determine the levels of miR-4732-3p in non-small cell lung cancer (NSCLC) tissue samples. A co-culture system was established to assess the release of miRNA via exosomes from NSCLC cells. RNA immunoprecipitation (RIP) and miRNA pull-down were applied to validate the interaction between miR-4732-3p and heterogeneous nuclear ribonucleoprotein K (hnRNPK) protein. Cell functional assays, cell derived xenograft, dual-luciferase reporter experiments, and western blot were applied to examine the effects of miR-4732-3p on MFSD12 and its downstream signaling pathways, and the impact of hnRNPK in NSCLC.

**Results:**

We enriched exosomes derived from NSCLC cells using the fucose-captured strategy and detected a significant upregulation of miR-4732-3p in fucosylated exosomes present in the serum, while its expression declined in NSCLC tissues. miR-4732-3p functioned as a tumor suppressor in NSCLC by targeting 3'UTR of MFSD12, thereby inhibiting AKT/p21 signaling pathway to induce cell cycle arrest in G2/M phase. NSCLC cells preferentially released miR-4732-3p via exosomes instead of retaining them intracellularly, which was facilitated by the interaction of miR-4732-3p with hnRNPK protein for selective sorting into fucosylated exosomes. Moreover, knockdown of hnRNPK suppressed NSCLC cell proliferation, with the elevated levels of miR-4732-3p in NSCLC tissues but the decreased expression in serum fucosylated exosomes.

**Conclusions:**

NSCLC cells escape suppressive effects of miR-4732-3p through hnRNPK-mediated sorting of them into fucosylated exosomes, thus supporting cell malignant properties and promoting NSCLC progression. Our study provides a promising biomarker for NSCLC and opens a novel avenue for NSCLC therapy by targeting hnRNPK to prevent the "exosome escape" of tumor-suppressive miR-4732-3p from NSCLC cells.

**Supplementary Information:**

The online version contains supplementary material available at 10.1186/s13046-024-03048-1.

## Introduction

Lung cancer, one of the most prevalent malignancies, has become the foremost cause of cancer-related deaths worldwide [1], and non-small cell lung cancer (NSCLC) accounts for approximately 82% of all lung cancer cases, comprising subtypes such as adenocarcinoma (LUAD), squamous cell cancers (LUSC), and large cell cancers [2]. NSCLC patients diagnosed at TNM stage I have been demonstrated to have a significantly higher five-year survival rate of up to 65%, compared with 5% at TNM stage IV [3]. Therefore, it is of paramount importance to improve patient outcomes by identifying biomarkers for diagnosis and elucidating the mechanisms driving NSCLC progression.

Exosomes, a specific type of extracellular vesicles with a diameter ranging from 40–160 nm, contain a lipid bilayer structure protecting the cargo from degradation [4]. Various types of cells have been shown to secrete exosomes with diverse cargo to obtain material exchange and cellular communication [5], of which microRNAs (miRNAs) have been well defined to be abundant in tumor-derived exosomes and participate in the development of many malignancies [6, 7]. Especially mentioned, tumor-derived exosomal miRNAs can be released by cancer cells into the serum [8], thereby providing potential non-invasive biomarkers for NSCLC diagnosis, as well as promising molecular targets for NSCLC therapy.

Previous studies have identified certain tumor-associated glycan chains aberrantly expressed in exosomes derived from cancer cells, serving as key signatures of malignant transformation [9, 10]. Fucosylation, one of the most common glycosylation modifications, is closely associated with the development of malignancies [11, 12]. Thus, it is reasonable to propose that capturing serum fucosylated exosomes and analyzing miRNA profiles might offer a promising approach for diagnosis and understanding the mechanisms underlying the sorting of miRNAs during the progression of NSCLC.

In our pilot study, we observed an elevated expression of miR-4732-3p in serum fucosylated exosomes from early LUAD patients, in comparison to both healthy controls (HCs) and patients with benign pulmonary nodules (BPNs) utilizing miRNA sequencing and qRT-PCR [13]. Nevertheless, the potential role of serum fucosylated exosomal miR-4732-3p in NSCLC progression remains unclarified and needs to be elucidated.

In this study, we first utilized the fucose-captured strategy, based on lentil lectin-magnetic beads with an affinity for fucose on the exosome membrane, to enrich tumor-derived exosomes. Our results showed that the upregulation of miR-4732-3p in serum fucosylated exosomes from NSCLC patients, and its suppressive effects on NSCLC cells. Moreover, our study sheds light on the unique behavior of NSCLC cells to escape suppressive effects of miR-4732-3p through hnRNPK-mediated selective sorting into fucosylated exosomes, supporting cell malignant properties and thus promoting NSCLC progression.

## Results

### miR-4732-3p is highly expressed in serum fucosylated exosomes but downregulated in tumor tissues of NSCLC patients

**Fig. 1:**
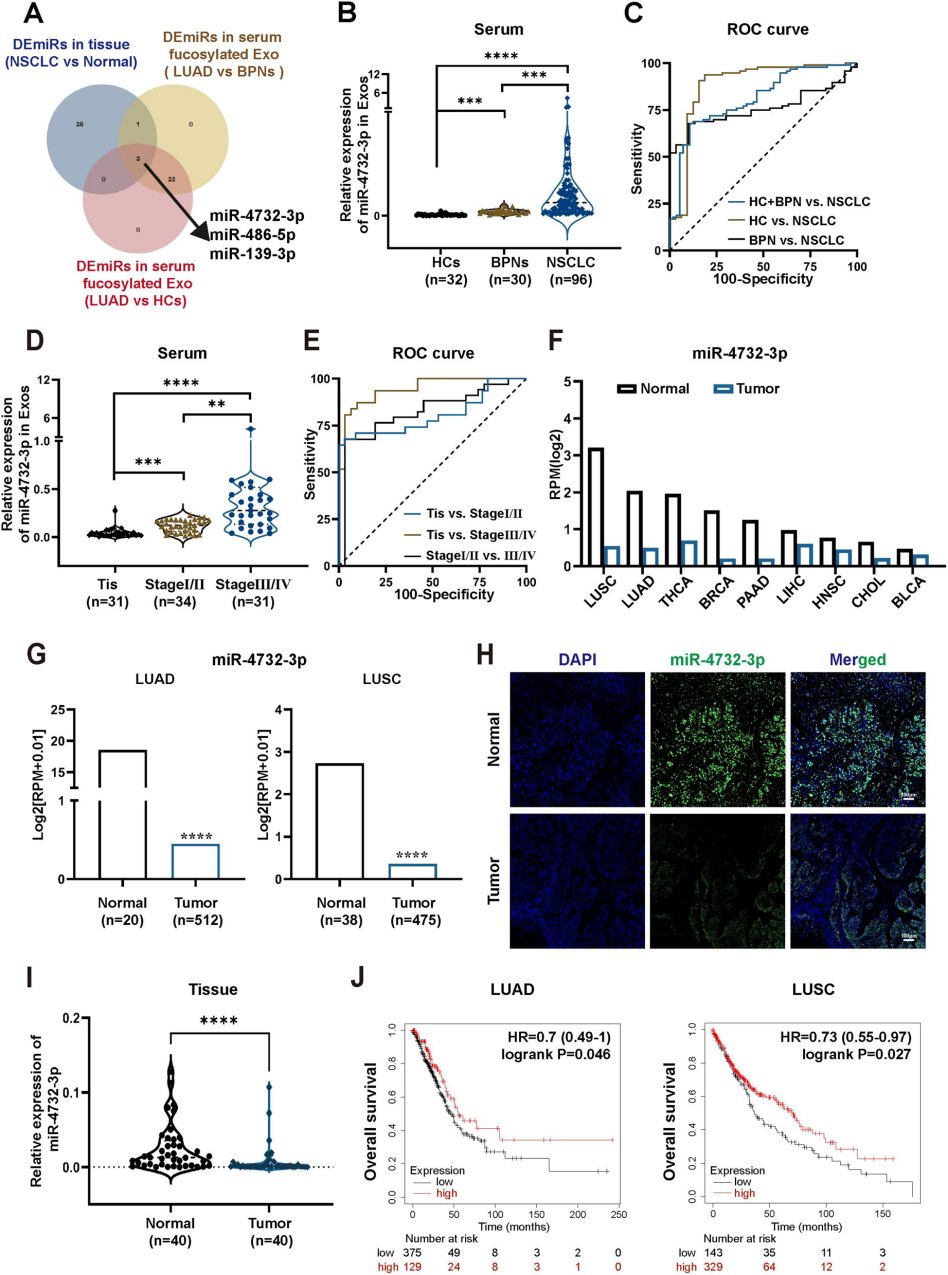
miR-4732-3p is highly expressed in serum fucosylated exosomes but downregulated in NSCLC tissues. **A** Venn diagram exhibiting the overlap of DEmiRs in serum fucosylated exosomes from early LUAD patients compared with both BPNs and HCs groups, together with DEmiRs in NSCLC tissues. **B** qRT-PCR was performed to determine miR-4732-3p levels in serum fucosylated exosomes from NSCLC patients (*n* = 96), BPNs (*n* = 30), and HCs (*n* = 32). **C** ROC curve analysis of serum fucosylated exosomal miR-4732-3p for diagnosing NSCLC patients form BPNs and HCs. **D** Levels of miR-4732-3p were detected via qRT-PCR in serum fucosylated exosomes from NSCLC patients at diverse stages: Tis (*n* = 31), Stage I/II (*n* = 34), and Stage III/IV (*n* = 31). **E** The ability of serum fucosylated exosomal miR-4732-3p to discriminate NSCLC patients at diverse stages was evaluated using ROC curve analysis. **F-G** Expression of miR-4732-3p in cancerous tissues according to the GEDs (**F**) and ENCORI (**G**) databases. **H-I** miR-4732-3p levels in NSCLC tissues were evaluated by the fluorescence intensity via FISH (**H**) and relative expression via qRT-PCR (**I**) analysis. **J** Kaplan–Meier survival analysis of NSCLC patients according to hsa-miR-4732 expression. Data are shown as the mean ± SD from at least three independent experiments. ^**^p < 0.01; ^***^p < 0.001; ^****^p < 0.0001; ns, not significant

To discover potential miRNAs participating in intercellular communication and NSCLC progression, we conducted a comprehensive analysis. Initially, we reanalyzed miRNA sequencing data from serum fucosylated exosomes and identified differentially expressed miRNAs (DEmiRs) in the serum fucosylated exosomes from early LUAD patients compared with both the healthy controls (HCs) and patients with benign pulmonary nodules (BPNs). We then identified DEmiRs (|logFC|> 2 and *p* < 0.05) in NSCLC via TCGA database analysis. By intersecting these three datasets, three candidate miRNAs (miR-4732-3p, miR-486-5p, and miR-139-3p) stood out (Fig. 1A). Considering that miR-486-5p and miR-139-3p have been extensively studied in various malignancies [14, 15], but the role of miR-4732-3p remains largely unknown, we therefore explored the expression patterns of miR-4732-3p and potential effects of miR-4732-3p on NSCLC progression.

We first investigated the expression of serum fucosylated exosomal miR-4732-3p in NSCLC patients, BPNs, and HCs. Our findings revealed that miR-4732-3p exhibited significantly higher levels in serum fucosylated exosomes from NSCLC patients, compared with both BPN and HC individuals (Fig. 1B). Subsequently, we determined the diagnostic value of serum fucosylated exosomal miR-4732-3p for NSCLC using receiver operating characteristic (ROC) curve analysis and calculated area under the curve (AUC), sensitivity, specificity, and predictive values (Table 1). Especially mentioned, we noticed that AUC was 0.823 (0.758–0.889) when discriminating NSCLC patients from HC and BPN groups, thereby substantiating its diagnostic potential (Fig. 1C). Meanwhile, the levels of serum fucosylated exosomal miR-4732-3p were increased concomitantly with advanced-stage NSCLC (Fig. 1D). More importantly, our analysis of ROC curves for serum fucosylated exosomal miR-4732-3p in discriminating different stages of NSCLC patients demonstrated AUC values of 0.823 (0.715–0.930) for distinguishing NSCLC patients in situ from those at stage I/II, 0.808 (0.694–0.923) for differentiating patients at stage I/II from those at stage III/IV, and 0.946 (0.894–0.998) for discriminating between NSCLC patients in situ and those at stage III/IV (Fig. 1E). Furthermore, the correlation analysis of the clinical characteristics of NSCLC patients revealed a positive association between the expression of serum fucosylated exosomal miR-4732-3p and TNM stages (Table 2). Interestingly, both the GEDs and ENCORI databases indicated that miR-4732-3p was dramatically downregulated in NSCLC tissues compared with that in adjacent non-tumor tissues (Fig. 1F and G). Further fluorescence in situ hybridization (FISH) and qRT-PCR analysis both confirmed the aberrant downregulation of miR-4732-3p in NSCLC tissues with weaker fluorescence intensity and relative expression (Fig. 1H and I). Taken together, miR-4732-3p was found to be significantly elevated in serum fucosylated exosomes but decreased in the cancerous tissues of NSCLC patients. Notably, our findings suggest its potential capacity to impede NSCLC progression with an association between the expression of miR-4732 and the lower hazard ratio in the predominant subtypes of NSCLC (Fig. 1J). Additional evidence of other malignancies also demonstrated a positive correlation between the expression of miR-4732 and improved prognosis for these cancer patients (Fig. S1). Consequently, we hypothesized that miR-4732-3p might confer a protective role by the suppression of NSCLC process.

**Table 1:** The diagnostic efficacy evaluation of serum fucosylated exosomal miR-47323-3p for NSCLC

Serum fucosylated exosomal miR-47323-3p	AUC-ROC (95% CI)	Sensitivity% (95% CI)	Specificity% (95% CI)	Positive predictive value%	Negative predictive value%
HC + BPN vs. NSCLC	0.823 (0.758–0.889)	67.71 (57.83–76.22)	90.32 (80.45- 95.49)	91.55	64.37
HC vs. NSCLC	0.886 (0.798–0.969)	90.63 (83.13–94.99)	84.38 (68.25–93.14)		
BPN vs. NSCLC	0.759 (0.679–0.840)	67.71 (57.83–76.22)	90.00 (74.38–96.54)		
Tis vs. Stage I/II	0.823 (0.715–0.930)	67.66 (0.84 -80.87)	96.77 (83.81–99.83)		
Tis vs. Stage III/IV	0.946 (0.894–0.998)	83.87 (67.37–92.91)	93.55 (79.28–98.85)		
Stage I/II vs. Stage III/IV	0.808 (0.694–0.923)	67.74 (50.14–81.43)	97.06 (85.08- 99.85)

**Table 2:** Relationship between the serum fucosylated exosomal miR-47323-3p levels and clinicopathological characteristics

Parameter	Number		Serum fucosylated exosomal miR-47323-3p		*χ*^*2*^	*P*-value
	Number	%	< Median	≥ Median		
**Age (years)**						
< 60	73	0.76	38	35	3.283	0.07
≥ 60	23	0.24	7	16		
**Gender**						
Male	70	0.73	46	24	3.033	0.082
Female	26	0.27	12	14		
**Type**^**a**^						
LUAD	51	0.53	22	29	0.016	0.901
LUSC	43	0.45	18	25		
**Tumor invasion**						
T0-2	62	0.65	37	25	6.558	0.010^*^
T3-4	34	0.35	11	23		
**Lymph node metastasis**						
N0	45	0.47	29	16	7.069	0.008^**^
N1-3	51	0.53	19	32		
**M classification**						
M0	68	0.71	45	23	24.401	< 0.0001^****^
M1	28	0.29	3	25		
**TNM Stage**						
0, I/II	65	0.68	44	21	25.201	< 0.0001^****^
III/IV	31	0.32	4	27		

^a^Two cases of large cell lung cancer were not included in the analysis of lung cancer subtype classification

^*^p < 0.05

^**^p < 0.01

^****^p < 0.0001

### miR-4732-3p is a tumor-suppressive miRNA in vitro and in vivo

**Fig. 2:**
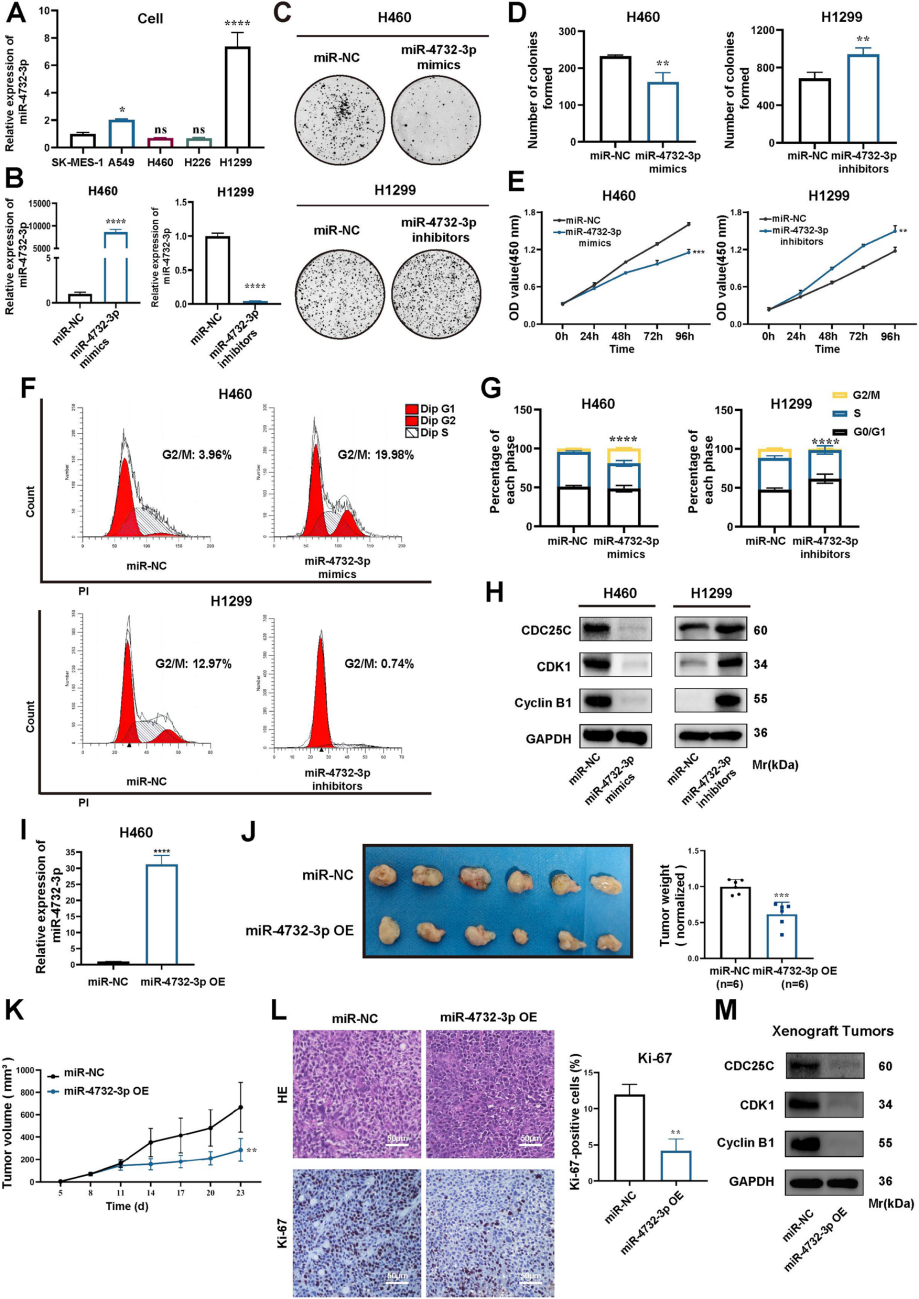
miR-4732-3p inhibits the proliferation of NSCLC in vitro and in vivo. **A** qRT-PCR analysis of miR-4732-3p expression in NSCLC cell lines. **B** qRT-PCR was performed to examine miR-4732-3p expression in NSCLC cells transfected with miR-4732-3p mimics and inhibitors. **C-E** Colony formation assays (**C-D**), and CCK8 assays (**E**) were performed to evaluate the proliferation ability of NSCLC cells transfected with miR-4732-3p mimics and inhibitors. **F-G** Cell cycle assays were conducted to examine the effect of miR-4732-3p mimics and inhibitors on NSCLC cell cycle progression. **H** Western blot analysis was used to detect the expression of G2/M phase-related protein in NSCLC cells transfected with miR-4732-3p mimics and inhibitors. **I** qRT-PCR was performed to assess the expression of miR-4732-3p in H460 cells following infection with lentivirus engineered to overexpress miR-4732-3p (miR-4732-3p OE) and negative control (miR-NC). **J** The indicated H460 cells (miR-NC and miR-4732-3p OE) were injected into nude mice and xenograft tumors were harvested. Representative images and measurement of tumor weight in xenograft tumors were shown (*n* = 6). **K** Measurement and analysis of tumor volume in xenograft tumors with H460 cells stably overexpressing miR-4732-3p and miR-NC. **L** Histological examination of xenograft tumors by hematoxylin and eosin (HE) staining and Ki-67 expression levels examined by immunohistochemistry (IHC) (Scale bars = 50 μm). **M** Western blot analysis investigating the impact of miR-4732-3p on G2/M phase-related protein in vivo. Data are shown as the mean ± SD from at least three independent experiments. ^*^p < 0.05; ^**^p < 0.01; ^***^p < 0.001; ^****^p < 0.0001; ns, not significant

To reveal the role of miR-4732-3p in NSCLC cells, we conducted qRT-PCR to assess its expression in several NSCLC cell lines including SK-MES-1, A549, H460, H226, and H1299. We found that H460 and H1299 expressed the lowest and highest miR-4732-3p, respectively (Fig. 2A). In order to determine the impact of miR-4732-3p on behaviors of NSCLC cells, we transfected H460 cells with miR-4732-3p mimics to overexpress it, but transfected H1299 cells with miR-4732-3p inhibitors to knock it down (Fig. 2B).

Colony formation assays revealed a 30% decrease in colony numbers for H460 cells transfected with miR-4732-3p mimics compared to that in the control group transfected with non-targeting miR-NC (Fig. 2C and D). Similarly, overexpression of miR-4732-3p led to a reduction of absorbance values from 1.60 to 1.15 in CCK8 assay, indicating a decreased number of viable H460 cells (Fig. 2E). Collectively, the results from both colony formation and CCK8 assays underscore the pivotal role of miR-4732-3p in inhibiting the proliferation of NSCLC cells. We also performed phalloidin staining and transwell assays to determine the effects of miR-4732-3p on migration and invasion ability of NSCLC cells. Phalloidin staining assay displayed that NSCLC cells overexpressing miR-4732-3p became rounder and smaller, whereas NSCLC cells with miR-4732-3p knockdown exhibited notable morphological changes and more filopodia (Fig. S2A), which have been proven to be crucial for tumor invasion [16]. Also, transwell assay results suggested a decrease in the number of migratory and invasive H460 cells due to miR-4732-3p overexpression, whereas its depletion led to the opposite trend (Fig. S2B).

To elucidate the underlying mechanisms of miR-4732-3p in suppressing NSCLC cells proliferation, we performed a transcriptome sequencing on H460 cells with miR-4732-3p mimics and miR-NC and identified differentially expressed genes (DEGs) for subsequent Gene Ontology (GO) analysis (Fig. S3A). GO analysis revealed that the DEGs were primarily associated with the G2/M phase of cell cycle (Fig. S3B). Consistently, the results of cell cycle assays showed that cell cycle was arrested at the G2/M phase in NSCLC cells with miR-4732-3p overexpression, but accelerated at the G2/M phase in NSCLC cells with miR-4732-3p knockdown (Fig. 2F and G). Moreover, the expression of G2/M phase-related protein, including cell division cycle 25C (CDC25C), cyclin dependent kinase 1 (CDK1), and CyclinB1, was noticeably decreased and increased in NSCLC cells with miR-4732-3p overexpression and knockdown, respectively (Fig. 2H), corroborating the role of miR-4732-3p in G2/M phase arrest.

To evaluate the potential role of miR-4732-3p in vivo, we established a xenograft model by injecting nude mice with H460 cells stably overexpressing miR-4732-3p and miR-NC (Fig. 2I). Our findings demonstrated that miR-4732-3p significantly impeded tumor growth, as evidenced by a substantial decrease in tumor weight (Fig. 2J), reduced tumor growth rate (Fig. 2K), and decreased Ki-67 expression within the tumors (Fig. 2L). In alignment with our hypotheses, western blot analysis of the xenograft tumors further corroborated the inhibitory effect of stable miR-4732-3p overexpression on the expression of G2/M phase-related proteins (Fig. 2M). Taken together, these results suggest that miR-4732-3p hinders NSCLC progression by inducing G2/M phase arrest both in vitro and in vivo.

### NSCLC cells preferentially release miR-4732-3p into exosomes

The theory of "exosome escape" states that cancer cells must secrete suppressive circRNAs to maintain cancer cell fitness [17]. Consistent with it, we observed that miR-4732-3p, a tumor-suppressive miRNA, was abundantly expressed in serum fucosylated exosomes from NSCLC patients. We further investigated the secretion ability of NSCLC cells via exosomes for different miRNAs, including miR-4732-3p, as well as two tumor-promoting miRNAs, miR-92b-3p and miR-1180-3p [18, 19]. These two miRNAs were found to be highly expressed in serum fucosylated exosomes from early-stage LUAD patients in our preliminary study [13]. Additionally, these two miRNAs have been elucidated to enhance the proliferation of NSCLC cells (Fig. S4A and B).

**Fig. 3:**
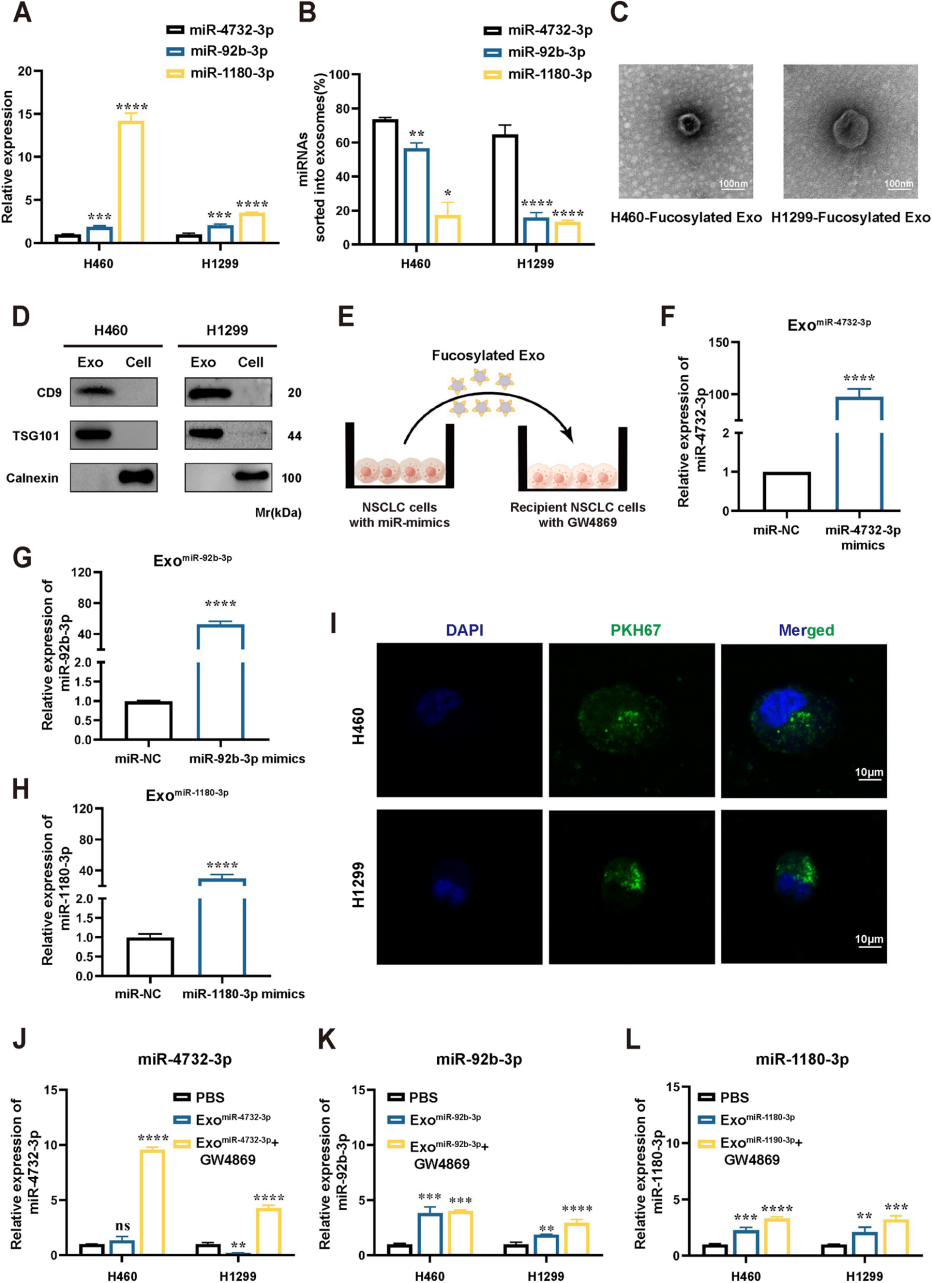
NSCLC cells preferentially release miR-4732-3p into exosomes. **A** Relative expression of miR-4732-3p, miR-92b-3p, and miR-1180-3p in NSCLC cells. **B** NSCLC cells was treated with GW4869 (10 μM) to assess the effects of exosome secretion on intracellular miRNA expression via qRT-PCR analysis and the proportion of each miRNA sorted into exosomes was calculated. **C** Images of fucosylated exosomes derived from NSCLC cells were photographed by transmission electron microscopy (TEM). Scale bar = 100 nm. **D** Western blot analysis was performed to detect typical exosome markers, including TSG101, CD9, and Calnexin (negative control). **E** Graphic illustration of co-culture system, in which we isolated fucosylated exosomes enriched with miRNA and added them into conditioned medium (CM) of NSCLC cells. **F–H** Fucosylated exosomes enriched with miR-4732-3p (**F**), miR-92b-3p (**G**), and miR-1180-3p (**H**) were obtained following transfecting NSCLC cells with miRNA mimics. **I** Internalization of PKH67-labeled fucosylated exosomes (green) by NSCLC cells. Scale bar = 10 μm. **J-L** NSCLC cells were co-cultured with blank control (PBS), or exosomes rich in miR-4732-3p, miR-92b-3p, and miR-1180-3p. We further added GW4869 (10 μM) to the co-culture system and analyzed the expression of intracellular miRNA in NSCLC cells co-cultured with exosomes under conditions where exosome secretion was suppressed, but exosome uptake was allowed. qRT-PCR was applied to verify the expression of miR-4732-3p (**J**), miR-92b-3p (**K**), and miR-1180-3p (**L**) in NSCLC cells after co-culturing. Data are shown as the mean ± SD from at least three independent experiments. ^*^p < 0.05; ^**^p < 0.01; ^***^p < 0.001; ^****^p < 0.0001; ns, not significant

To determine the proportion of miRNAs secreted into exosomes, we first examined the levels of miR-4732-3p, miR-92b-3p, and miR-1180-3p in NSCLC cells (Fig. 3A) and evaluated their expression following treatment with GW4869 (10 μM) to hinder exosome secretion. Our results showed that a greater proportion of miR-4732-3p was released extracellularly via exosomes, compared to miR-92b-3p and miR-1180-3p (Fig. 3B). Moreover, we isolated fucosylated exosomes derived from NSCLC cells and validated their presence through transmission electron microscopy (Fig. 3C), western blot (Fig. 3D), and nanoparticle tracking analysis (Fig. S5B). Next, as depicted in Fig. 3E, we separately co-cultured fucosylated exosomes enriched with miR-4732-3p, miR-92b-3p, or miR-1180-3p (Fig. 3F-H) with NSCLC cells, and confocal microscopy revealed that exosomes were internalized by the NSCLC cells and localized around the nucleus (Fig. 3I and S4C). Furthermore, we examined the expression of intracellular miR-4732-3p in NSCLC cells that took up fucosylated exosomes enriched with miR-4732-3p using qRT-PCR, demonstrating that the expression of intracellular miR-4732-3p did not increase following co-culturing. However, the intracellular expression of miR-4732-3p significantly upregulated in NSCLC cells treated with GW4869 (Fig. 3J), suggesting the secretion ability of NSCLC cells for miR-4732-3p via exosomes. Furthermore, our data revealed that co-culturing with fucosylated exosomes enriched with miR-92b-3p or miR-1180-3p both led to higher intracellular levels of these two tumor-promoting miRNAs (Fig. 3K and L). Overall, these findings support that NSCLC cells preferentially release miR-4732-3p via exosomes rather than retaining it intracellularly, in accordance with the "exosome escape" hypothesis.

### Heterogeneous nuclear ribonucleoprotein K (hnRNPK) mediates selective sorting of miR-4732-3p into fucosylated exosomes

**Fig. 4:**
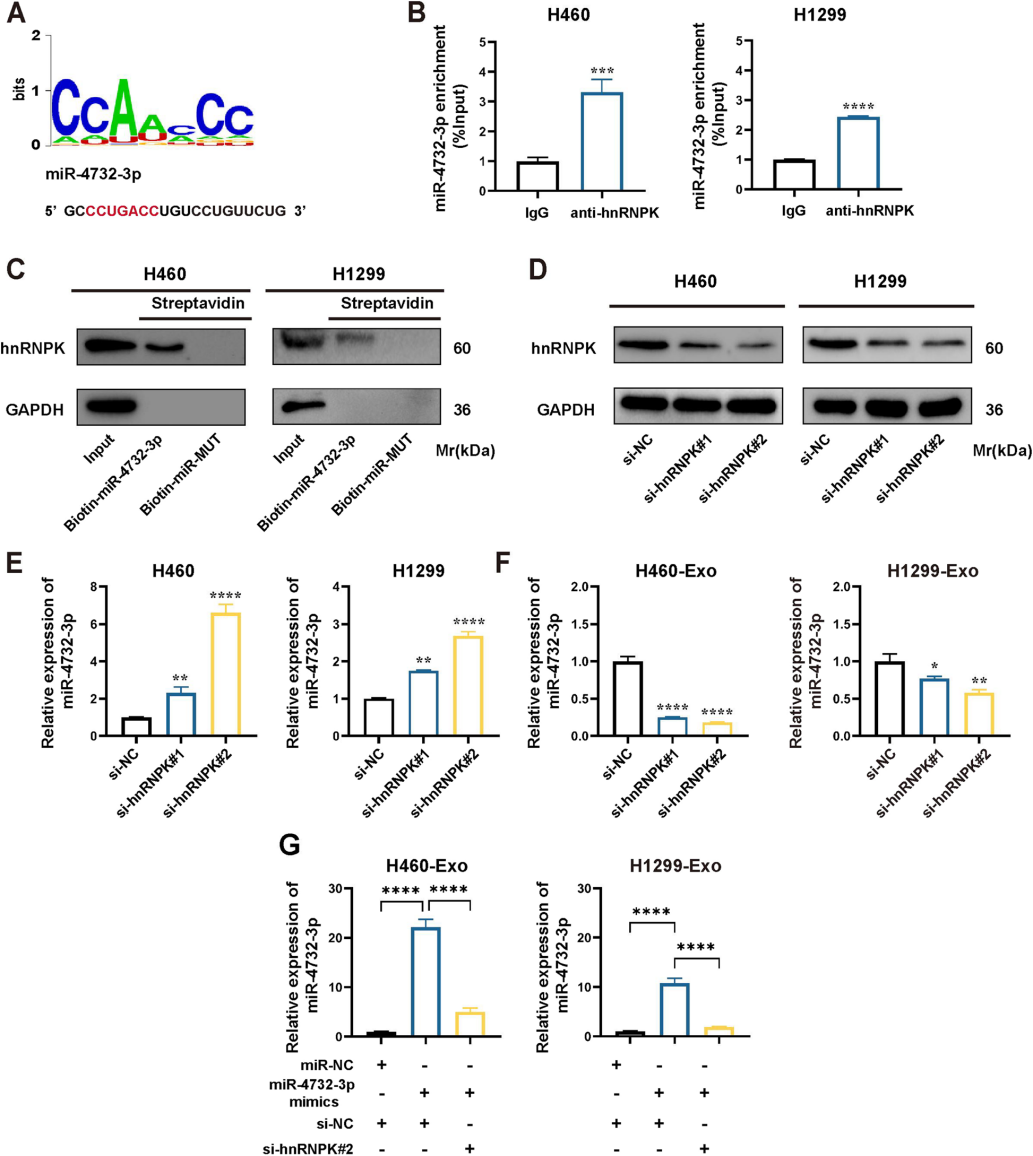
miR-4732-3p is sorted into fucosylated exosomes via hnRNPK. **A** Sequence motifs of hnRNPK binding site predicted by RBPsuite. **B** RIP assay using hnRNPK antibody and qRT-PCR were performed, anti-IgG as the negative control. **C** miRNA pull-down and western blot analysis were conducted to confirm interaction between miR-4732-3p and hnRNPK protein. **D** Western blot analysis was applied to define knockdown efficiency of hnRNPK in NSCLC cells transfected with si-hnRNPK. **E–F** qRT-PCR analysis of miR-4732-3p levels in NSCLC cells (**E**), and their corresponding fucosylated exosomes (**F**) following hnRNPK knockdown. **G** qRT-PCR analysis was performed to determine the effects of miR-4732-3p mimics and si-hnRNPK on the levels of fucosylated exosomal miR-4732-3p. Data are shown as the mean ± SD from at least three independent experiments. ^*^p < 0.05; ^**^p < 0.01; ^***^p < 0.001; ^****^p < 0.0001; ns, not significant

Specific motifs in miRNAs have been identified to influence their cellular retention or allocation into exosomes by interacting with RNA-binding proteins (RBPs) [20]. By utilizing the RBPsuite website to predict motifs, we discovered a potential binding sequence between hnRNPK and miR-4732-3p (Fig. 4A). Previous studies have reported that hnRNPK, a member of the hnRNP protein family, might play an important role in sorting specific RNAs into exosomes [21]. In order to validate this interaction, we conducted RNA immunoprecipitation (RIP) and miRNA pull-down assays to confirm the enrichment of miR-4732-3p using the hnRNPK antibody (Fig. 4B) and the binding ability of miR-4732-3p with hnRNPK protein (Fig. 4C), respectively. To investigate the involvement of hnRNPK in sorting miR-4732-3p into fucosylated exosomes, we conducted knockdown experiments targeting hnRNPK using siRNAs (Fig. 4D). Our results showed that hnRNPK knockdown led to increased levels of miR-4732-3p in NSCLC cells (Fig. 4E), but decreased expression of miR-4732-3p in fucosylated exosomes derived from NSCLC cells (Fig. 4F). Furthermore, we determined that NSCLC cells with miR-4732-3p overexpression secreted fucosylated exosomes with elevated levels of miR-4732-3p. However, hnRNPK knockdown could counteract the elevation effects of intracellular miR-4732-3p expression on exosomal miR-4732-3p (Fig. 4G), further indicating the involvement of hnRNPK in the sorting of miR-4732-3p into fucosylated exosomes.

### hnRNPK promotes NSCLC cell proliferation in vitro and in vivo

**Fig. 5:**
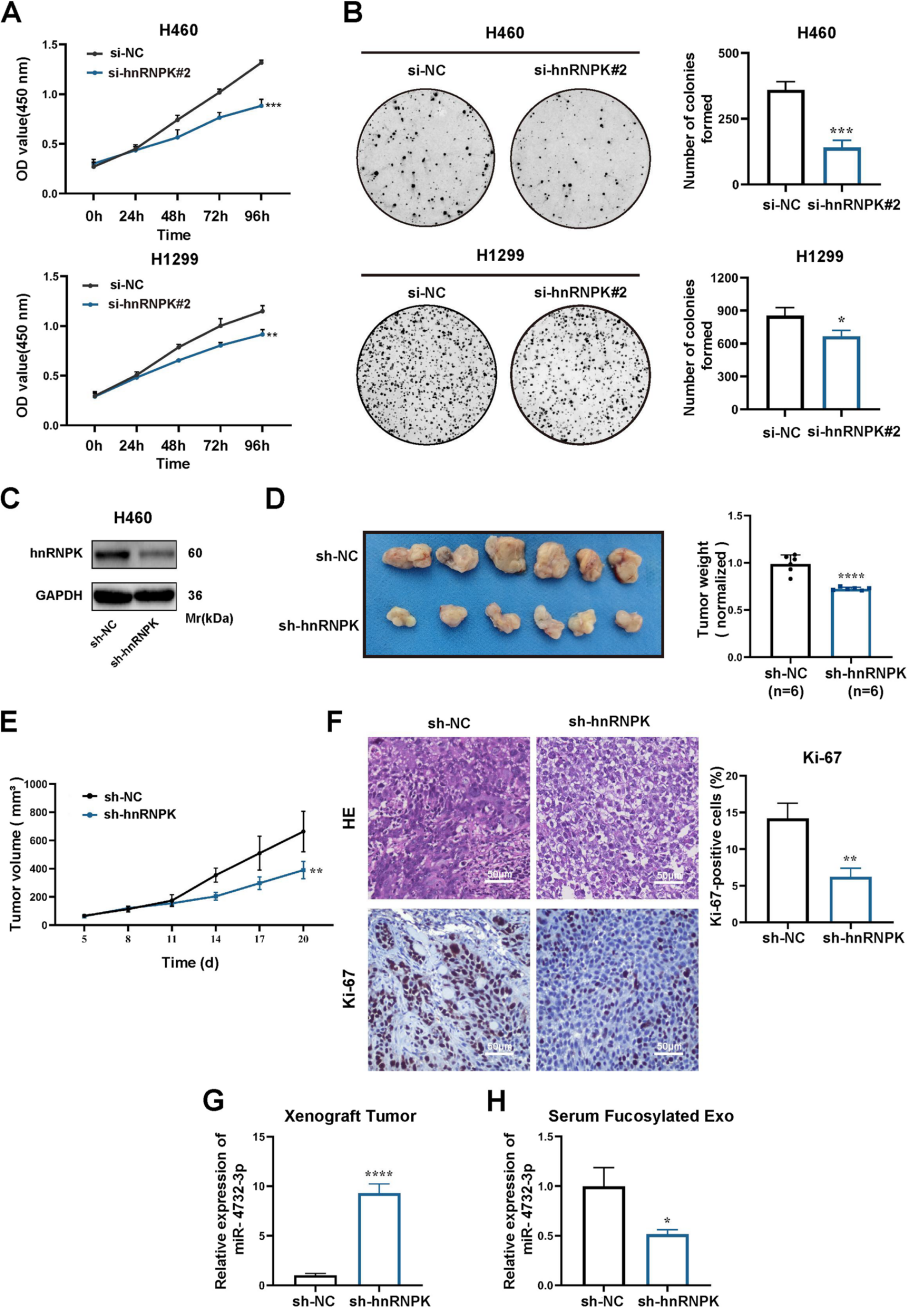
Knockdown of hnRNPK suppresses NSCLC in vitro and in vivo. **A-B** The proliferation ability of NSCLC cells transfected with hnRNPK siRNAs was evaluated through CCK8 **(A)** and colony formation assays (**B**). **C** H460 cells were infected with lentivirus engineered to knock down hnRNPK (sh-hnRNPK) and negative control (sh-NC). The protein expressions were analyzed by western blot. **D** The indicated H460 cells (sh-NC and sh-hnRNPK) were injected into nude mice and xenograft tumors were harvested. Representative images and measurement of tumor weight in xenograft tumors were shown (*n* = 6). **E** Measurement and analysis of tumor volume in xenograft tumors of sh-NC and sh-hnRNPK groups. **F** Histological examination of xenograft tumors through HE staining and analysis of Ki-67 expression levels by IHC (Scale bars = 50 μm). **G** qRT-PCR analysis of miR-4732-3p expression in xenograft tumor tissues with stably hnRNPK knockdown. **H** qRT-PCR analysis of fucosylated exosomal miR-4732-3p expression in serum from nude mice with stably hnRNPK knockdown. Data are shown as the mean ± SD from at least three independent experiments. ^*^p < 0.05; ^**^p < 0.01; ^***^p < 0.001; ^****^p < 0.0001; ns, not significant

Regarding the role of hnRNPK in NSCLC cells proliferation, we initially performed CCK8 (Fig. 5A) and colony formation assays (Fig. 5B), which demonstrated a inhibition of NSCLC cell proliferation and colony formation ability upon knockdown of hnRNPK. To further investigate the impact of hnRNPK in vivo, a xenograft model was established by injecting nude mice with H460 cells in which hnRNPK was stably knocked down (Fig. 5C). Our findings showed that the knockdown of hnRNPK suppressed tumor growth, as evident from a notable reduction in tumor weight (Fig. 5D), tumor growth rate (Fig. 5E), and Ki-67 expression in xenograft tumors (Fig. 5F). Regarding the the sorting of miR-4732-3p into fucosylated exosomes, we detected miR-4732-3p expression in xenograft tumor tissues and serum fucosylated exosomes via qRT-PCR. There were elevated levels of miR-4732-3p in xenograft tumor tissues (Fig. 5G), but declined miR-4732-3p expression in serum fucosylated exosomes from nude mice with hnRNPK knockdown (Fig. 5H). Together, these results suggest that hnRNPK promotes NSCLC progression by selectively sorting miR-4732-3p into fucosylated exosomes, which are subsequently released into circulation.

### miR-4732-3p induces G2/M arrest by regulating MFSD12/AKT/p21 axis in NSCLC cells

**Fig. 6:**
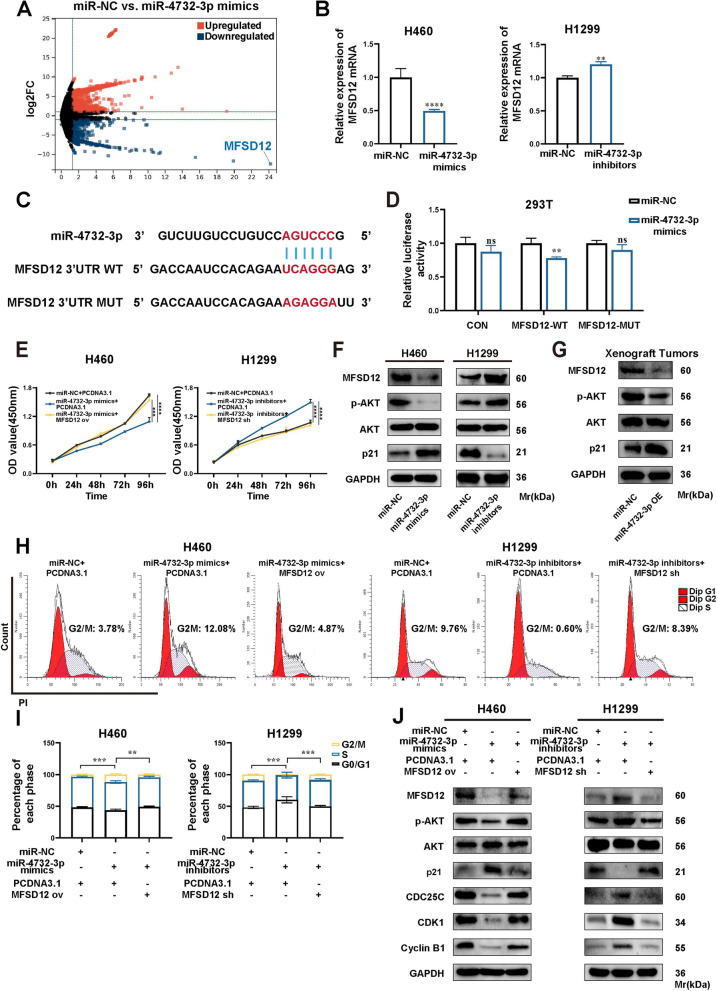
miR-4732-3p targets 3'UTR of MFSD12 in NSCLC cells. **A** Volcano plot presenting significantly differentially expressed genes (DEGs) (|log2FC|>1 and *p* < 0.05) between two groups: H460 cells transfected with miR-4732-3p mimics and miR-NC. **B** qRT-PCR analysis of MFSD12 mRNA expression in NSCLC cells following transfection of miR-4732-3p mimics and inhibitors. **C** Predicted binding sites between miR-4732-3p and the 3'UTR of MFSD12 mRNA. **D** Luciferase assay performed in 293T cells to validate the binding interaction between miR-4732-3p and MFSD12. **E** CCK8 assays examining the proliferation of NSCLC cells with overexpression or suppression of miR-4732-3p and MFSD12. **F-G** Western blot analysis of MFSD12 protein and the AKT/p21 signaling pathway in NSCLC cells after treatment as described above (**F**), and in xenograft tumors with miR-4732-3p overexpression (**G**). **H-I** Cell cycle analysis performed to assess the combined effects of miR-4732-3p and MFSD12 in NSCLC cells. **J** Western blot analysis conducted to investigate the AKT/p21 signaling pathway and G2/M phase-related protein after the indicated treatment. Data are shown as the mean ± SD from at least three independent experiments. ^*^p < 0.05; ^**^p < 0.01; ^***^p < 0.001; ^****^p < 0.0001; ns, not significant

miRNAs have been proven to regulate gene expression by binding to the 3'UTR of mRNA, thereby resulting in mRNA degradation or translation inhibition. These regulatory mechanisms play a pivotal role in maintaining the balance of gene expression and biological processes. To determine which genes are regulated by miR-4732-3p, we employed the TargetScan website to predict the target genes with the potential binding sites of miR-4732-3p. Among these genes, major facilitator superfamily domain containing 12 (MFSD12) attracted our attention, because MFSD12 showed a significant downregulation with the highest fold change in DEGs of transcriptome sequencing analysis on H460 cells with miR-4732-3p mimics and miR-NC (Fig. 6A) and MFSD12 has ever been reported to promote cancer cell proliferation [22]. qRT-PCR results also revealed that miR-4732-3p overexpression in H460 cells led to a significant decrease of MFSD12 mRNA, whereas miR-4732-3p knockdown in H1299 cells caused an increase of MFSD12 mRNA (Fig. 6B), suggesting that MFSD12 is the potential target of miR-4732-3p. To prove that MFSD12 is a direct target of miR-4732-3p, we generated reporter plasmids by inserting 3'UTR of MFSD12 with intact miR-4732-3p binding site (WT) or mutated miR-4732-3p binding site (MUT) to the 3' terminal of luciferase gene (Fig. 6C), and then performed dual-luciferase reporter assays in 293T cells. Our results showed that miR-4732-3p could reduce luciferase activity in the MFSD12 WT group, but had no effect on MUT group (Fig. 6D), providing evidence that miR-4732-3p directly targeted MFSD12 mRNA and inhibited the expression of MFSD12. Furthermore, the function of MFSD12 in NSCLC cells was determined through CCK8 assays, which showed that MFSD12 partially attenuated the inhibitory effects of miR-4732-3p on NSCLC cells proliferation (Fig. 6E), suggesting that miR-4732-3p suppresses NSCLC cells proliferation by targeting MFSD12.

Due to the downregulation of AKT expression observed in transcriptome sequencing results and previous studies highlighting the regulatory effects of the AKT/p21 pathway on cell cycle [23], our further investigation aimed to elucidate how MFSD12 regulates NSCLC progression via the AKT/p21 signaling pathway. We first performed western blot analysis to assess AKT/p21 expression, demonstrating decreased phosphorylation of AKT and significantly elevated p21 expression in H460 cells overexpressing miR-4732-3p (Fig. 6F). Likewise, western blot analysis of xenograft tumors also confirmed the effects of miR-4732-3p overexpression on the downregulation of protein expression in the AKT/p21 signaling pathway in vivo (Fig. 6G). Furthermore, cell cycle assays showed that the G2/M arrest induced by miR-4732-3p mimics in H460 cells could be alleviated by MFSD12 overexpression (Fig. 6H and I). Likewise, the reduced phosphorylation of AKT and the G2/M phase-related proteins were counteracted, and the elevated levels of p21 declined accordingly upon overexpression of both miR-4732-3p and MFSD12 in H460 cells (Fig. 6J), suggesting that MFSD12 enhances the AKT/p21 signaling pathway and contributes to cell cycle progression. Herein, we believe that intracellular miR-4732-3p induces G2/M arrest and suppresses NSCLC progression via MFSD12/AKT/p21 axis.

### MFSD12 is upregulated in NSCLC and associated with unfavorable prognosis

**Fig. 7:**
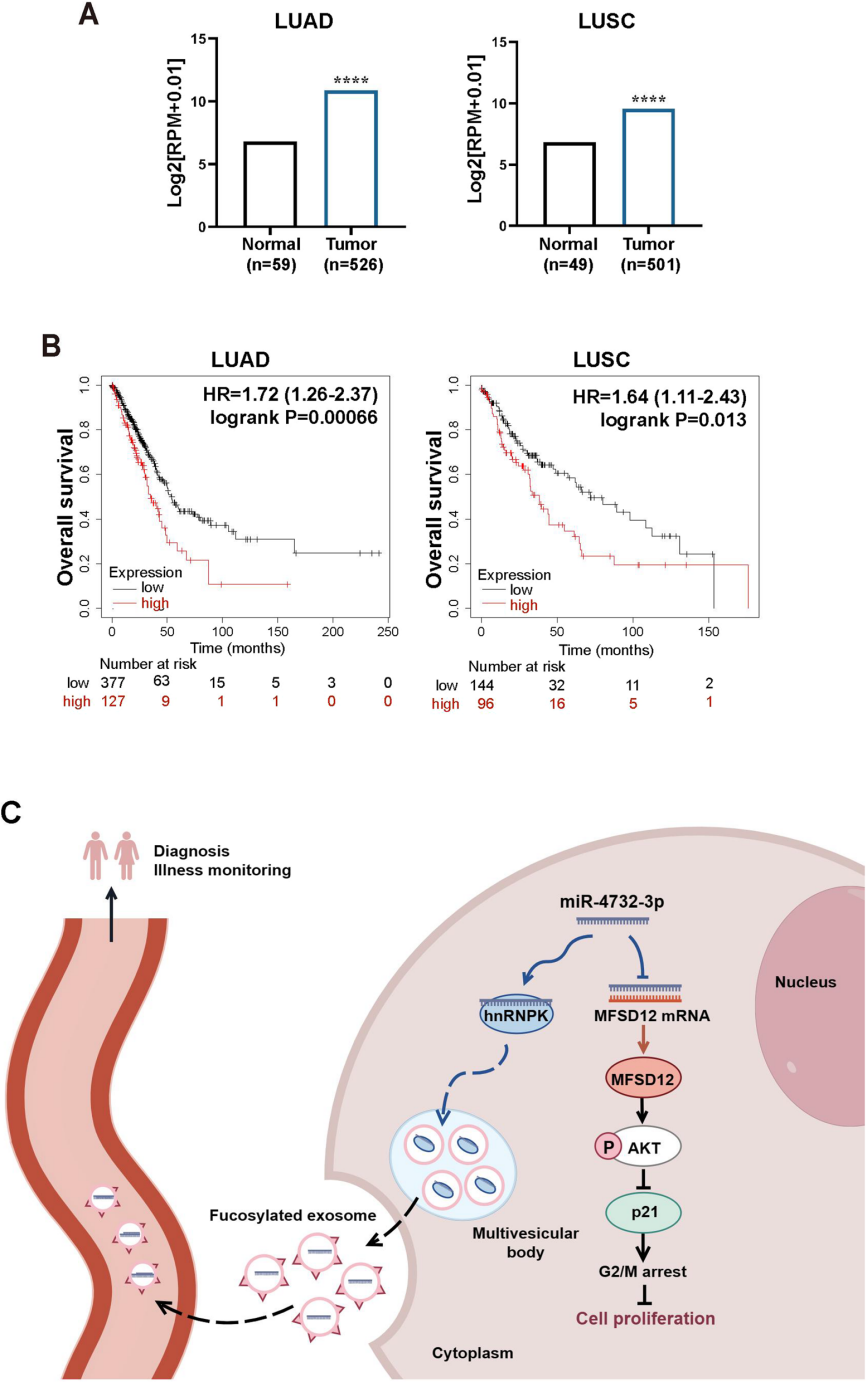
MFSD12 is upregulated in NSCLC and associated with unfavorable prognosis. **A** ENCORI database analysis depicting the MFSD12 mRNA expression in LUAD and LUSC tissues. **B** Kaplan–Meier analysis illustrating the negative relationship between MFSD12 expression and overall survival (OS) rates of LUAD and LUSC patients. **C** Proposed working model depicting the mechanism by which tumor-suppressive miR-4732-3p is sorted into fucosylated exosomes through its interaction with hnRNPK protein, thereby promoting NSCLC progression. Data are shown as the mean ± SD from at least three independent experiments. ^****^p < 0.0001

To further investigate MFSD12 mRNA expression in human NSCLC, we utilized the ENCORI database and found increased MFSD12 mRNA expression in both LUAD and LUSC tissues (Fig. 7A). Moreover, higher levels of MFSD12 in NSCLC tissues were associated with unfavorable outcomes, as observed in the Kaplan-Meier plotter website, which suggests its potential oncogenic role (Fig. 7B).

Based on the findings in this study, we proposed a working model: tumor-suppressive miR-4732-3p inhibits NSCLC progression by targeting MFSD12 to downregulate AKT signaling, which leads to p21-mediated G2/M cell cycle arrest; NSCLC cells avoid suppressive effects of miR-4732-3p through hnRNPK-mediated sorting of miR-4732-3p into fucosylated exosomes (Fig. 7C).

## Discussion

Exosomal miRNAs have emerged as essential roles in intercellular communication and tumor progression [24]. As a potent biological sample recommended for depicting pulmonary health conditions, bronchoalveolar lavage fluid (BALF) entails the invasiveness and patient discomfort during acquisition. Compared with BALF, blood sample presents the advantages of both noninvasion and readily accession. Moreover, there are currently consistent performances of biomarkers between BALF and blood sample. For example, miR-223 and miR-142 expressions have been reported to consistently upregulate in both BALF and sera during pneumonia [25]; another study revealed 10 cell cycle-regulatory biomarkers with similar expression patterns by analyzing and comparing exosomal miRNA profiles derived from BALF and plasma of Icotinib-resistant NSCLC patients [26]. Therefore, we opted to collect serum sample as an alternative access of BALF to explore NSCLC-related exosomal miRNAs, aiming to provide an optimal understanding for NSCLC progression by noninvasive liquid biopsy approach.

Especially mentioned, aberrantly high fucosylation in cancer cells usually results in an augmented release of fucosylated exosomes, a specific type of exosomes bearing fucose molecules on their surface, which might yield valuable insights into the occurrence and development of malignancies [27]. Our pilot study employed the fucose-captured strategy based on lentil lectin-magnetic beads to isolate serum fucosylated exosomes [13]. Ultracentrifugation (UC) is widely recognized as the "gold standard" and the most commonly employed method for exosome isolation [28]. In this study, we determined whether the fucose-capture strategy is preferable to the traditional UC for enriching exosomes derived from NSCLC cells. Initially, we isolated exosomes from cell conditioned medium (CM) of two NSCLC cells (H460 and H1299), as well as two normal cells (BEAS-2B and 293T), using both isolation methods, and then performed nanoparticle tracking analysis (NTA) to compare the sizes and concentration of exosomes. To determine the effectiveness of the fucose-captured strategy for capturing tumor-derived exosomes, we utilized the particle number ratio (Fucosylated Exo/UC Exo) as a quantitative measurement (Fig. S5A). Notably, we observed a higher ratio in the CM of NSCLC cells compared to normal cells, indicating the selective enrichment of tumor-derived exosomes through the fucose-captured strategy (Fig. S5). These findings suggest that capturing fucosylated exosomes is conducive to enriching tumor-derived exosomes and reliably depicting the miRNA profiles that reflect tumor progression. In our pilot study, the fucose-captured strategy, which was implemented to isolate serum exosomes derived from tumors, exhibited a promising potential of serum fucosylated exosomal miRNAs including miR-4732-3p for the early diagnosis of LUAD [13]. In this study, miR-4732-3p was demonstrated to be significantly upregulated in serum fucosylated exosomes from NSCLC patients while declined in NSCLC tissues. Notably, Kaplan–Meier plotter analysis revealed a positive correlation between the expression of miR-4732 and the overall survival (OS) rates of NSCLC patients. Thus, it is reasonable to deduce that miR-4732-3p, which is highly expressed in serum fucosylated exosomes from NSCLC patients, might act as a protective factor for NSCLC patients.

It is well documented that cancer cells could discard certain tumor-suppressive molecules, including circRNAs and miRNAs, through exosomes to maintain tumor development [29, 30], a process known as the "exosome escape" theory [17]. Analogously, we demonstrated that miR-4732-3p, released by NSCLC cells via exosomes, exhibited markedly suppressive effects on the proliferation of NSCLC cells by targeting MFSD12. MFSD12, involved in the import of cysteine into melanosomes and lysosomes [31, 32], has been reported to enhance melanoma cell proliferation [22]. However, its function in NSCLC remains undetermined. Herein, we elucidated the role of MFSD12 in promoting tumor growth, wherein its overexpression partially mitigated the suppressive effects of miR-4732-3p on NSCLC cells. It is currently acknowledged that AKT phosphorylation is initiated by a variety of upstream signals, notably the activation-induced stimulation of phospoinositide 3-kinase (PI3K) stemming from insulin/IGF receptors or the engagement with growth factors. Notably, several negative modulators, including phosphatase and tensin homolog (PTEN), mechanistic target of rapamycin complex 2 (mTORC2), and PH domain leucine-rich repeat protein phosphatase (PHLPP), have been documented to directly suppress AKT protein phosphorylation [33]. Moreover, there have been reports suggesting that ubiquitin-mediated degradation of heat shock protein 90 (Hsp90) can indeed promote AKT phosphorylation [34]. Our findings revealed that the miR-4732-3p-mediated downregulation of MFSD12 did not affect total AKT protein abundance; however, it significantly dampened the phosphorylation of the AKT protein. Consequently, MFSD12 protein seems to exert an indirect influence on AKT phosphorylation regulation. It's reasonable to deduce that the reduced expression of MFSD12 could hinder upstream AKT signaling cascades to cause a decline of AKT phosphorylation level, by potentially decreasing PI3K activity or augmenting the expression of negative regulators which will be defined in our future study. Furthermore, dysregulation of AKT is responsible for cancer cell survival, and the AKT/p21 signaling pathway serves as a regulatory mechanism of cell cycle progression at both the G1/S and G2/M checkpoints [35]. We initially disclosed G2/M arrest according to cell cycle results, along with a corresponding decrease in expression of G2/M-phase related proteins including CDK1 and CyclinB1, which were attributed to miR-4732-3p. Our further research results showed that the upregulated expression of p21 mainly caused the suppression of cyclin-dependent kinase activity during the G2/M phase. In conclusion, miR-4732-3p suppresses NSCLC cell proliferation and tumor growth by modulating the MFSD12/AKT/p21 signaling pathway and inducing G2/M arrest.

Exosomes have gained increasing attentions based on their cargo transportation and biological function [36, 37]. Current studies have stated that the levels of miRNAs in exosomes positively correlate with their expression in the originating cells, implying that miRNAs, highly expressed in cells, are likewise abundantly present in exosomes [5]. Nevertheless, mounting evidence has suggested that cancer cells exhibit a complex miRNA secretion profile. In addition to actively releasing highly expressed tumor-promoting miRNAs, they also discard tumor-suppressive miRNAs, which are believed to contribute to the maintenance and enhancement of malignant properties in cancer cells [38, 39]. In our pilot study, we identified certain upregulated serum fucosylated exosomal miRNAs associated with early diagnosis of LUAD, including a combination of tumor-promoting miRNAs and tumor-suppressive miRNAs [13]. However, the extent to which miRNAs with tumor-promoting or tumor-suppressive effects could be sorted into fucosylated exosomes remains unclear. Herein, we demonstrated that NSCLC cells preferentially secreted tumor-suppressive miR-4732-3p over tumor-promoting miRNAs, including miR-92b-3p and miR-1180-3p via exosomes. Moreover, a large quantity of fucosylated exosomal miR-4732-3p taken up by NSCLC cells did not give rise to intracellular miR-4732-3p levels, indicating the predominant extracellular release of miR-4732-3p via exosomes which serve as irreplaceable carriers in NSCLC cells. However, transfection with miR-4732-3p mimics disrupted the balance between intracellular uptake and sorting processes, causing a surge in the intracellular expression of miR-4732-3p. Furthermore, we observed that the expression of serum fucosylated exosomal miR-4732-3p exhibited an elevation with the development of NSCLC, indicating that NSCLC cells may discard a higher quantity of tumor-suppressive miR-4732-3p into circulation via fucosylated exosomes in order to evade suppressive effects of miR-4732-3p and ultimately foster NSCLC progression.

Accumulating evidence supports the involvement of RBPs in selectively sorting miRNAs into exosomes [40, 41]. RBPs have been shown to interact with intracellular miRNAs to form RNA-loaded RBPs which are subsequently recruited to the sites of exosomes budding and sorted into exosomes [5]. The hnRNPK protein, a member of the heterogeneous nuclear ribonucleoprotein (hnRNP) family of RNA-binding proteins, plays crucial roles in multiple facets of RNA metabolism, including mRNA precursor splicing, transcription regulation, and regulation of miRNA [42, 43]. It has also been recognized for its involvement in the sequence-dependent packaging of miRNAs into exosomes [21]. Our findings validated the interaction between hnRNPK and miR-4732-3p and revealed its role in facilitating the selective packaging of miR-4732-3p into fucosylated exosomes for extracellular release. These results underscore the critical involvement of hnRNPK in the "exosome escape" of tumor-suppressive miR-4732-3p, unveiling a pivotal target for NSCLC therapy.

In summary, these findings shed light on the significant involvement of miR-4732-3p in NSCLC progression. Intracellularly, miR-4732-3p induces G2/M arrest and inhibits proliferation of NSCLC cells by targeting 3'UTR of MFSD12, thereby inhibiting AKT/p21 signaling pathway. In contrast, NSCLC cells preferentially secrete miR-4732-3p via hnRNPK into fucosylated exosomes and discard it extracellularly instead of retaining it intracellularly so as to escape tumor-suppressive effects of miR-4732-3p. Additionally, the knockdown of hnRNPK causes elevated levels of miR-4732-3p in NSCLC tissues and decreased levels of serum fucosylated exosomal miR-4732-3p, thereby suppressing NSCLC cells proliferation. Moreover, a significant amount of miR-4732-3p, discarded by NSCLC cells, is released into the bloodstream via fucosylated exosomes, resulting in a notable increase in the levels of serum fucosylated exosomal miR-4732-3p during NSCLC progression, thus highlighting its potential as a novel serum biomarker for the diagnosis and monitoring of NSCLC. Consequently, further investigation is warranted to elucidate the mechanisms underlying the selective sorting of additional tumor-suppressive miRNAs into fucosylated exosomes, and to devise a strategy targeting hnRNPK to prevent the "exosome escape" of tumor-suppressive miR-4732-3p from NSCLC cells, thereby opening up a new avenue for improving the five-year survival rates of NSCLC patients.

## Materials and methods

### Patients samples and cell lines

Human serum samples were obtained from 96 patients diagnosed with NSCLC, 30 patients with BPN (benign pulmonary nodule), and 32 HCs (healthy controls) at Fujian Provincial Hospital between January 2022 and December 2022. All serum samples were preserved at a temperature of -80℃ following centrifugation. In addition, tissue samples were collected from a total of 40 NSCLC patients, all of whom were histologically confirmed [44]. Written informed consent has been obtained and the study was approved by the Ethics Committee of Fujian Provincial Hospital (approval number: K2018-12–040/02, from January 2019 to December 2023).

Human NSCLC cell lines, including A549, H460, H226, H1299, and SK-MES-1, along with human normal lung epithelial cells (BEAS-2B) and human renal epithelial cells (293T), were all purchased from Procell (Wuhan, China). Both H460 and H1299 cells were maintained in Roswell Park Memorial Institute (RPMI) 1640 medium (Gibco, USA), enriched with 10% fetal bovine serum (FBS) sourced from Procell (Wuhan, China). Meanwhile, A549, H226, SK-MES-1, BEAS-2B, and 293T cells were cultured in Dulbecco's modified Eagle's medium (DMEM) (Gibco, USA), also supplemented with 10% FBS. All cell lines were authenticated through short tandem repeat profiling and were cultured strictly at 37℃ in a cell culture incubator with a controlled environment of 5% CO_2_.

### Fluorescence in situ hybridization (FISH)

The specific fluorescently labeled miR-4732-3p FISH probes were designed and synthesized by Servicebio (Wuhan, China), and the experiment was conducted following the manufacturer's instructions. All images were acquired on Nikon A1Si Laser Scanning confocal microscope (Nikon Instruments Inc., Japan).

### Cell transfection and stable cell lines

miR-4732-3p mimics and inhibitors, hnRNPK small interfering RNAs (siRNAs), and pcDNA3.1-MFSD12 overexpression and knockdown plasmids were designed and constructed by Zolgene (Fuzhou, China). EntransterTM-R4000 from Engreen (Beijing, China) was obtained to transfect miR-4732-3p mimics and inhibitors, and hnRNPK siRNAs. EntransterTM-H4000 Transfection reagent (Engreen) was applied to transfect plasmids as per instructions. After 24 h of transient transfection, the cells were harvested for subsequent cell functional assays.

To establish NSCLC cells with stable overexpression or suppression of miR-4732-3p and hnRNPK, lentiviral plasmids were constructed by Hanbio (Shanghai, China). Following lentiviral infection at an appropriate multiplicity of infection (MOI) value for 72 h and selection with puromycin, stably transfected NSCLC cells were obtained. Additionally, qRT-PCR and western blot analysis were conducted to assess the efficiency of transfection and infection. All sequences against specific targets are provided in the Supplementary Tables.

### Cell proliferation assays

Colony formation assay and CCK8 assay were carried out to assess the proliferation of NSCLC cells with transient transfection. For colony formation assay, cells were placed at 6-well plates and allowed to grow for weeks. Subsequently, the developed colonies were fixed, stained and counted using a light microscope. Additionally, cell viability was evaluated with cell counting kit-8 (CCK8) according to instructions (Cellcook, China). At specific time points, the CCK8 solution was added into wells and the absorbance was measured at 450 nm (OD450) using a microplate reader following a 1-h of incubation.

### Cell cycle assay

To synchronize the cell cycle, NSCLC cells were incubated with nocodazole (100 nM) for 14 h, effectively leading to their arrest at the G2-M phase transition point. Subsequently, cell cycle assay was conducted to determine the distribution of each cell cycle phase in NSCLC cells with transient transfection. Cells were first thoroughly washed with precooled PBS, subsequently fixed with 75% ethanol and incubated overnight at 4℃. On the following day, after centrifugation and another round of washing with PBS, the cell pellets were collected. The staining working solution containing propidium iodide (PI) and RNase A was then prepared (Meilunbio, China). The harvested cell pellets were resuspended in the freshly prepared staining working solution and incubated in the dark at 37℃ for 30 min. Upon completion of the staining process, cells were subjected to flow cytometry analysis for cell cycle determination, and the percentages of cells in each phase were quantified using ModFit LT software version 5.0.

### Transwell assays

Cell migration and invasion were assessed using 8.0 µm 24-well transwell plates (Corning, USA). The upper chamber was seeded with 50,000 cells, transfected with miR-4732-3p mimics and inhibitors, in RPMI 1640 medium, whereas the bottom chamber was full of medium containing 12% FBS. After a 48-h incubation, cells that invaded the membrane or migrated to the bottom chamber were fixed in 4% paraformaldehyde (PFA), stained with 0.1% crystal violet, and counted in five randomly selected microscope fields. Of note, the upper surface of the membrane was coated with 60 µL of Matrigel (BD Biosciences, USA) for the invasion assay.

### Phalloidin staining

After fixed in 4% PFA, NSCLC cells transfected with miR-4732-3p mimics and inhibitors were permeabilized using 0.1% Triton X-100 and subsequently blocked with 5% bovine serum albumin (BSA) in phosphate buffered saline (PBS) for 30 min. Next, staining for F-actin and DAPI was done to visualize cell structures, followed by images acquisition using confocal microscopy.

### Exosome isolation and validation

Exosomes from serum samples and cells CM were isolated using the fucose-captured strategy which bases on the specific affinity of lentil lectin-magnetic beads to fucosylated exosomes according to standard protocols [13]. Similarly, exosomes in cells CM were also isolated through ultracentrifugation (UC) as described in Figure S5A. To visualize the obtained exosomes using transmission electron microscopy (TEM), they were fixed and placed onto grids coated with copper mesh Formvar; 2% phosphotungstic acid was added to the grids for staining, and the exosomes were observed under a JEOL TEM (model JEM1230, Japan). Additionally, the concentration and size distribution of obtained exosomes were assessed using Nanoparticle Tracking Analysis (NTA) with ZetaView (Particle Metrix, Germany) and the obtained data were then analyzed using the accompanying software, ZetaView 8.04.02.

### Co-culture and exosome uptake assay

A co-culture assay was conducted to investigate the trafficking of exosomal miRNA derived from NSCLC cells. We first transfected NSCLC cells with miR-4732-3p mimics and isolated their secreted exosomes, thus obtaining exosomes enriched with miR-4732-3p. Using this method, we also obtained exosomes that are enriched with miR-92b-3p or miR-1180-3p. To monitor exosome uptake, exosomes obtained from cell CM were labeled with the PKH67 fluorescent cell linker and suspended in PBS following careful isolation. Subsequently, stained exosomes were incubated with NSCLC cells and observed using confocal fluorescence microscopy after staining cell nuclei with DAPI. During co-culture of NSCLC cells, GW4869 (10 µM, Cayman Chemical, USA), an inhibitor of exosome secretion, was also utilized to evaluate the role of exosome in releasing miR-4732-3p extracellularly.

### RNA extraction and qRT-PCR

To extract total RNA from exosomes, we used the miRNeasy® Mini Kit (Qiagen, Germany). Subsequent qRT-PCR was performed using reagent kits from Tiangen (Beijing, China).

For the extraction of total RNA from NSCLC cells, NucleoZOL (Takara, Japan) was utilized following a standard protocol. Similarly, qRT-PCR was performed using reagent kits from Takara. The primers synthesized by Shangya (Fuzhou, China) were listed in the Supplementary materials.

### Western blot

Total protein was extracted and protein concentrations were measured using protein assay kits from Solarbio (Beijing, China). The following procedures for western blot were performed: total protein was separated and transferred to a PVDF membrane. After blocking the membrane with 5% BSA, we incubated the membrane with various primary antibodies overnight at 4℃ and then with secondary antibodies. Finally, protein detection was performed using a chemiluminescence instrument.

### Transcriptome sequencing

We prepared H460 cells with miR-4732-3p overexpression and miR-NC (*n* = 3). RNA was extracted and used for illumina sequencing technology. The data generated was analyzed using the online platform from Majorbio for further analysis.

### Public data and bioinformatics analysis

We analyzed miRNA sequencing data with Sequence Read Archive (SRA) accession number PRJNA847004, consisting of serum fucosylated exosomes from eight HCs, eight BPNs, and eight early LUAD patients. miRNA-seq data specific to NSCLC tissues was obtained from cBioPortal (http://www.cbioportal.org/public-porta) to identify DEmiRs in NSCLC tissues. As for the levels of miR-4732-3p and MFSD12 in malignancies, GEDs (http://bioinfo.life.hust.edu.cn/web/GEDS/) and ENCORI (https://rnasysu.com/encori/index.php) databases were applied. Moreover, we assessed the correlation between miR-4732/MFSD12 expression and OS rates of patients with LUAD and LUSC, using the Kaplan–Meier plotter (http://kmplot.com). To predict binding sites between MFSD12 and miR-4732-3p, we used TargetScan (http://www.targetscan.org/vert_80/), a database that provides information on miRNA-target interactions. In addition, we investigated RBPs that potentially bind to miR-4732-3p by utilized RBPsuite website (www.csbio.sjtu.edu.cn/bioinf/RBPsuite/), which offers tools for predicting potential protein-RNA interactions.

### Dual-luciferase reporter assay

Dual-luciferase reporter plasmids, consisting of MFSD12 wild-type (WT), mutant (MUT), and control (CON), were constructed in psicheck2.0 vectors (Zolgene, China). 293T cells were co-transfected with reporter plasmids and miR-4732-3p mimics using EntransterTM-H4000 and EntransterTM-R4000 transfection reagents from Engreen. Cell lysates were obtained to perform luciferase reporter assays following transfection for 24 h.

### RNA immunoprecipitation (RIP) assay

Cell lysates were immunoprecipitated with magnetic beads conjugated with hnRNPK antibody using the PureBinding Kit (Geneseed, China), with IgG (ab205718, Abcam) as negative control. Finally, the captured RNA were detected by qRT-PCR.

### miRNA pull-down assay

We utilized the miRNA-protein pull-down kit from Geneseed to validate the interaction between hnRNPK and miR-4732-3p. Biotinylated miR-4732-3p and its mutant sequences were incubated with the cell lysates to facilitate binding with their respective interacting proteins. Finally, the eluted protein from the RNA–protein complex was determined based on the results obtained from western blot analysis.

### Animal experiments

Specific pathogen-free BALB/c nude mice, aged four to six weeks, were randomly divided into two groups (*n* = 6), and performed subcutaneous injections of 5 × 10^6^ cells in 100 μL serum-free RPMI 1640 and Matrigel (1:1) to establish cell line-derived xenograft (CDX) models. One group of mice received subcutaneous injections with H460 cells stably overexpressing miR-4732-3p (miR-4732-3p OE group) while the other group received H460 cells with only vectors (miR-NC group). CDX models were also developed using H460 cells, wherein hnRNPK was stably suppressed (sh-hnRNPK group) or with only vectors (sh-NC group). Tumor size was measured every 3 days and calculated using the formula: (length × width^2^) × 0.5 after measuring the length and width with the vernier caliper. Three weeks after tumor implantation, the mice were sacrificed for subsequent experiments. Consequently, all xenograft tumors were fixed, embedded, and stained with hematoxylin and eosin (HE).

Besides the aforementioned experiments, total RNA from xenograft tumor tissues and serum fucosylated exosomes of nude mice in sh-hnRNPK and sh-NC groups were extracted for further analysis. All animal experiments have been approved by the Animal Ethics Committee of Fujian Ambri Biotechnology Co., Ltd (approval number: IACUC FJABR 2022101101, from October 2022 to October 2023).

### Immunohistochemistry (IHC)

All paraffin sections of xenograft tumor tissues were deparaffinized, rehydrated, and then incubated with Ki-67 antibody after blocking. The following day, images were collected and analyzed following incubation with the secondary antibody and staining with DAB and hematoxylin. As for calculating the Ki-67 staining results, we counted 200 cells in each high-power field of view and recorded the number of Ki-67-positive cells (brown). The percentage obtained by dividing the number of Ki-67-positive cells by the total counted cells is the Ki-67-positive cells (%). Take the average of five fields of view as the Ki-67 expression level of the sample.

### Statistics analysis

Data are shown as mean ± standard deviation (SD) from at least three independent experiments. Statistical analysis was performed using GraphPad Prism 9.3. The Student's t-test was employed to assess differences between groups, while one-way analysis of variance (ANOVA) was utilized for analysis involving three or more groups. ROC curve and AUC value were determined to measure the performance of the diagnostic model. Relationship between serum fucosylated exosomal miR-4732-3p expression and clinicopathological feature was analyzed by Chi-square test. The cumulative OS rates were calculated using the Kaplan–Meier method, and significance was evaluated with the log-rank test. A significance level of *p* < 0.05 was considered statistically significant, unless specially noted otherwise.

### Supplementary Information

**Supplementary Material 1. **

**Supplementary Material 2. **

## Data Availability

All data pertinent to this study are contained within the article or can be obtained from the corresponding author upon a reasonable request.

## Abbreviations

NSCLC: Non-small cell lung cancer
LUAD: Lung adenocarcinoma
LUSC: Lung squamous cell cancers
miRNAs: MicroRNAs
MFSD12: Major facilitator superfamily domain containing 12
hnRNPK: Heterogeneous nuclear ribonucleoprotein K
BPN: Benign pulmonary nodule
HC: Healthy control
qRT-PCR: Real-time quantitative PCR
DEmiRs: Differentially expressed miRNAs
DEGs: Differentially expressed genes
ROC: Receiver operating characteristic
AUC: Area under the curve
FISH: Fluorescence in situ hybridization
OS: Overall survival
NC: Negative control
CDC25C: Cell division cycle 25C
CDK1: Cyclin dependent kinase 1
CM: Conditioned medium
UC: Ultracentrifugation
TEM: Transmission electron microscopy
NTA: Nanoparticle Tracking Analysis
Exo: Exosome
Fucosylated Exo: Fucosylated exosome
UC Exo: Ultracentrifugation exosome
RBP: RNA-binding protein
RIP: RNA immunoprecipitation
PI3K: Phospoinositide 3-kinase
PTEN: Phosphatase and tensin homolog
mTORC2: Mechanistic target of rapamycin complex 2
PHLPP: PH domain leucine-rich repeat protein phosphatase
Hsp90: Heat shock protein 90
FBS: Fetal bovine serum
RPMI: Roswell Park Memorial Institute
DMEM: Dulbecco's modified Eagle's medium
