# Supplementary material for: Tumor-suppressive miR-4732-3p is sorted into fucosylated exosome by hnRNPK to avoid the inhibition of lung cancer progression
Source: J Exp Clin Cancer Res. 2024 Apr 23;43:123. doi: 10.1186/s13046-024-03048-1 (PMC11036635; doi:10.1186/s13046-024-03048-1)
Supplement: Supplementary file 1 — Supplementary Material 1. [file 13046_2024_3048_MOESM1_ESM.pdf]

Figure S1

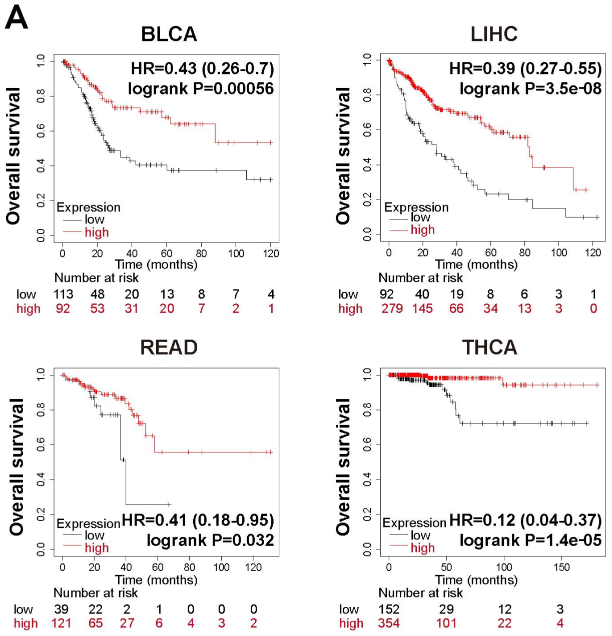

Fig. S1 Kaplan-Meier survival analysis results reveal positive correlations between miR-4732 expression and overall survival rates in bladder urothelial carcinoma (BLCA), liver hepatocellular carcinoma (LIHC), rectal adenocarcinoma (READ), and thyroid cancer (THCA).

Figure S2

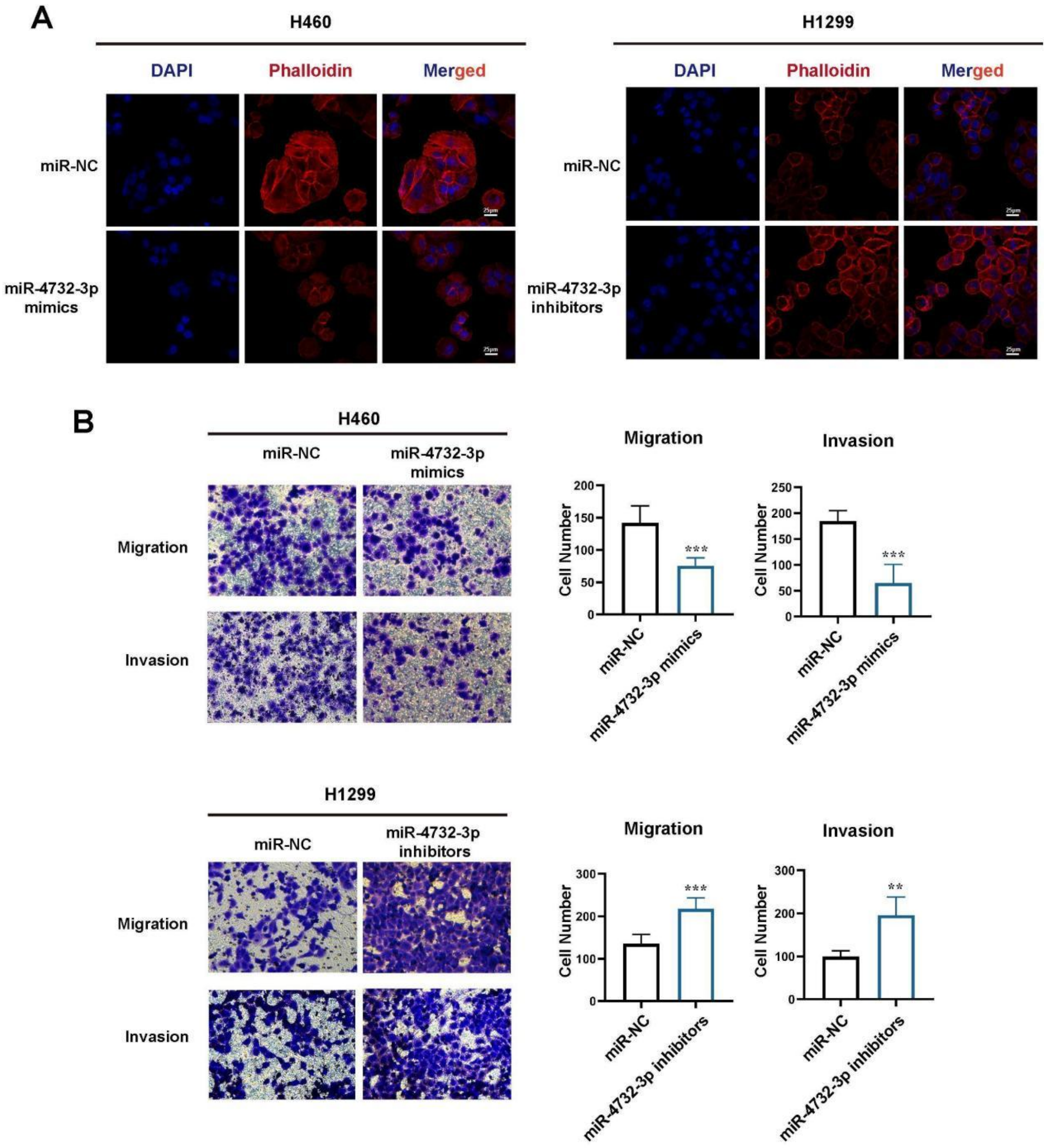

Fig. S2 miR-4732-3p inhibits the migration and invasion of NSCLC cells. (A) Representative images of filopodia staining performed in NSCLC cells were displayed. Scale bars = 25μm. (B) Migration and invasion capacities of NSCLC cells transfected with miR-4732-3p mimics and inhibitors were verified by transwell assays, 200×. Data are shown as the mean ± SD from at least three independent experiments. *$p < 0.05$; **$p < 0.01$; ***$p < 0.001$; ns, not significant.

Figure S3

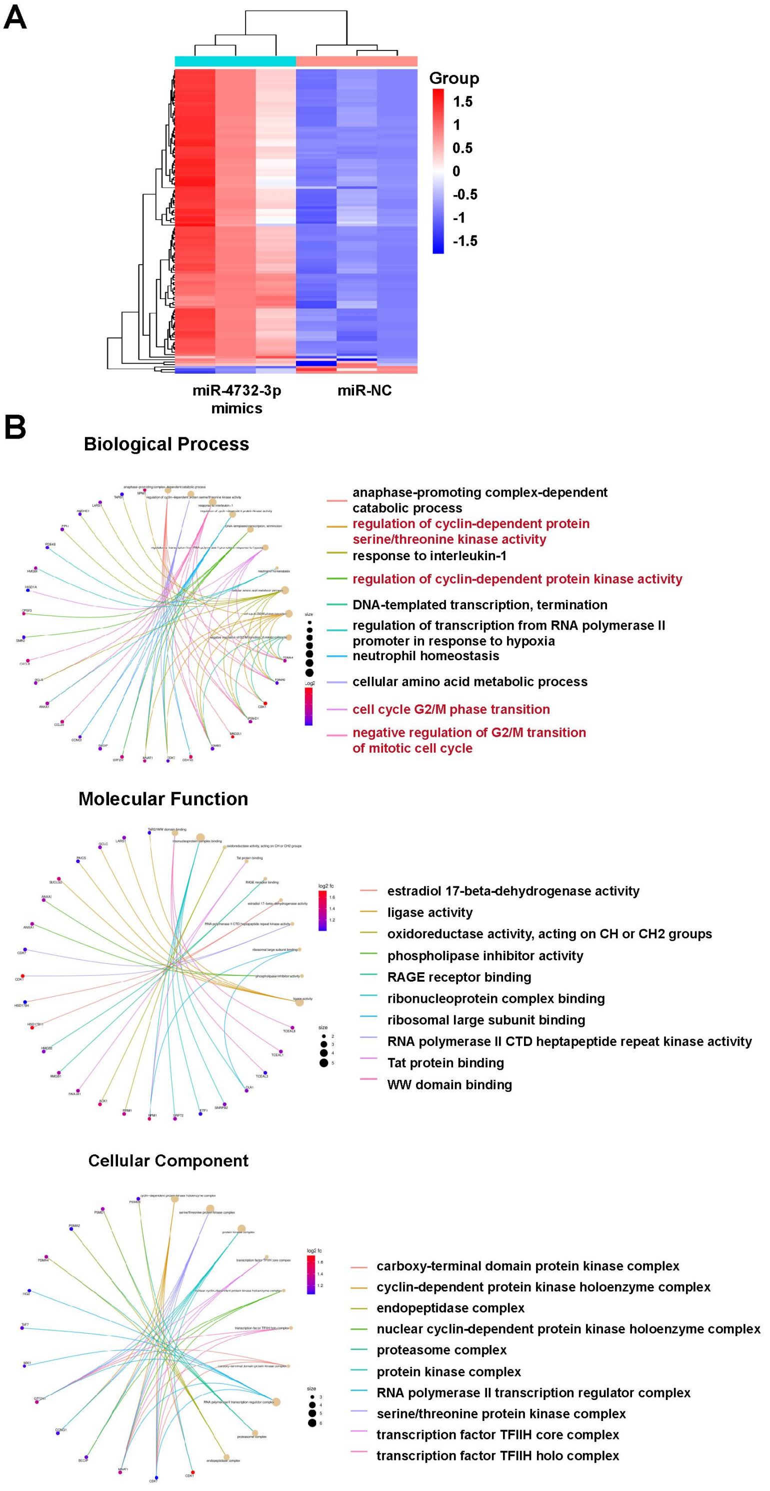

Fig. S3 (A) Heat map presenting significantly differentially expressed genes (DEGs) between two groups: H460 cells transfected with miR-4732-3p mimics and miR-NC. (B) Cnetplot displaying GO enrichment results of biological process, cellular component, and molecular function.

Figure S4

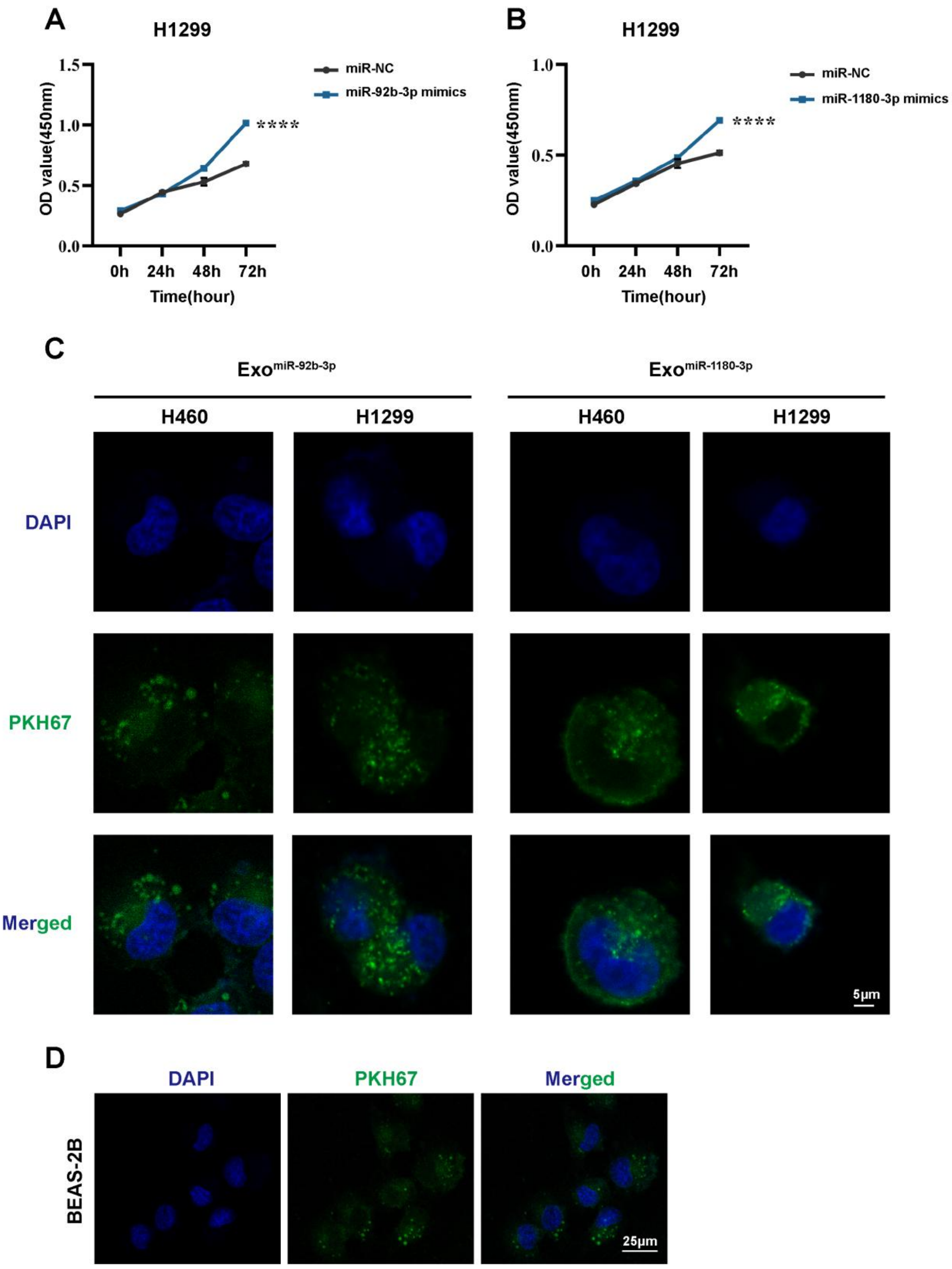

Fig. S4 (A) CCK8 assays were applied to determine the effects of miR-92b-3p and (B) miR-1180-3p on NSCLC cells proliferation. (C) Internalization of PKH67-labeled fucosylated exosomes by NSCLC cells. Scale bar = 5 µm. (D) Internalization of PKH67-labeled fucosylated exosomes by normals cells. Scale bar = 25 µm. Data are shown as the mean ± SD from at least three independent experiments. *p < 0.05; **p < 0.01; ***p < 0.001; ****p <0.0001; ns, not significant.

Figure S5

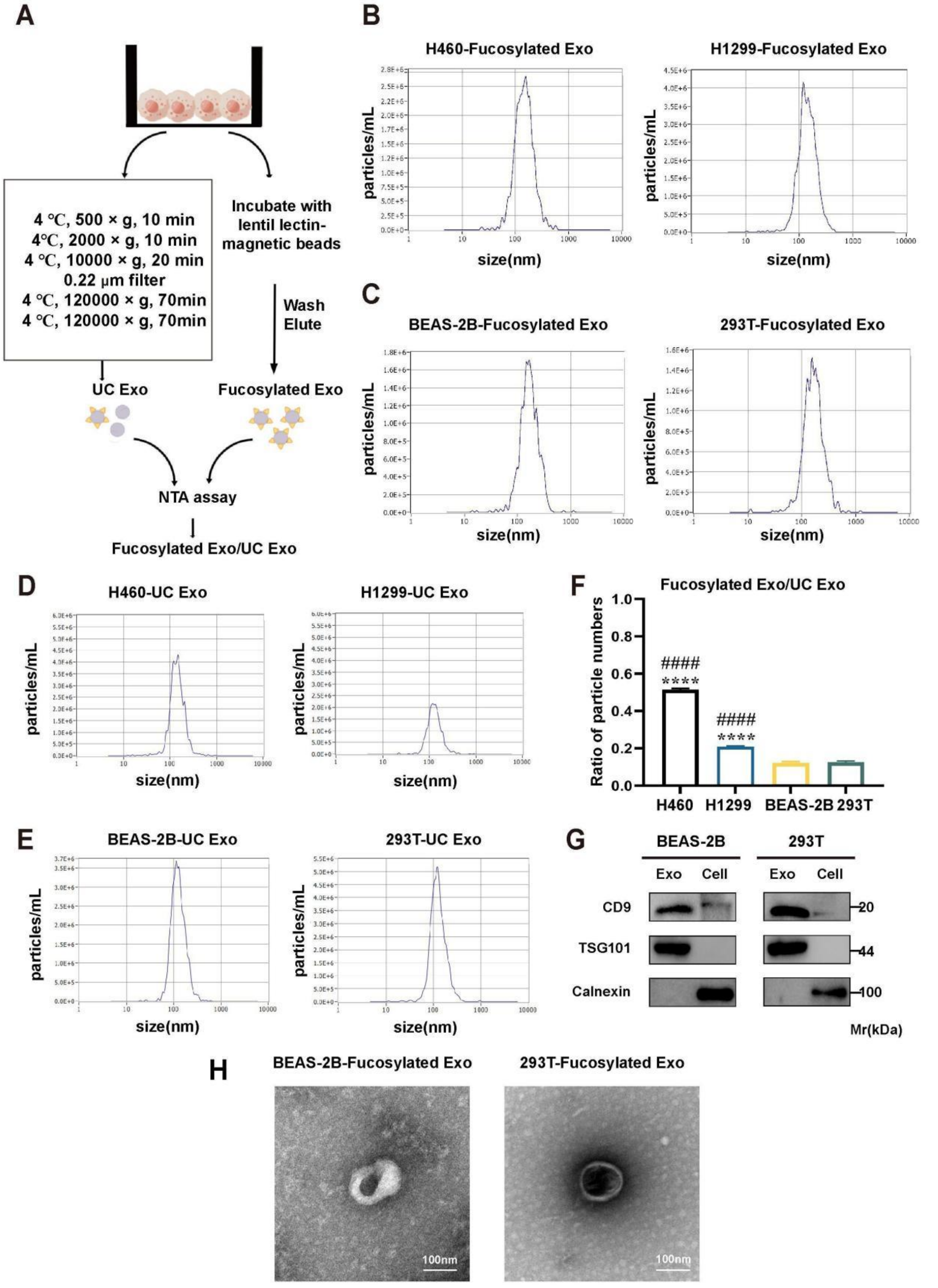

Fig. S5 Fucose-captured strategy enriches more fucosylated exosomes derived from NSCLC cells compared to normal cells. (A) Graphic illustration depicting isolation methods of exosomes derived from cells, alongside their subsequent experiment. (B) Concentration and size distribution of fucosylated exosomes derived from NSCLC cells and (C) normal cells were determined by NTA. (D) Concentration and size distribution of UC Exo derived from NSCLC cells and (E) normal cells were determined by NTA. (F) The ratio of particle numbers obtained from the

fucose-captured strategy and ultracentrifugation was analyzed to evaluate the effectiveness for capturing tumor-derived exosomes. (G) Western blot analysis was performed to detect typical exosome markers, including TSG101, CD9, and negative control, Calnexin. (H) Images of fucosylated exosomes were photographed by TEM. Scale bar = 100nm. Data are shown as the mean ± SD from at least three independent experiments. ****p <0.0001 versus BEAS-2B; #### p <0.0001 versus 293T.
