# Supplementary material for: Tumor-suppressive miR-4732-3p is sorted into fucosylated exosome by hnRNPK to avoid the inhibition of lung cancer progression
Source: J Exp Clin Cancer Res. 2024 Apr 23;43:123. doi: 10.1186/s13046-024-03048-1 (PMC11036635; doi:10.1186/s13046-024-03048-1)
Supplement: Supplementary file 2 — Supplementary Material 2. [file 13046_2024_3048_MOESM2_ESM.zip › Table S1 Sequences.docx]

**Table S1. Sequences for siRNA, miRNA mimics, and primers for qRT-PCR**

| **Sequences for siRNA, miRNA mimics and primers for qRT-PCR** |
| --- |
| GAPDH forward: 5′- AGGTGAAGGTCGGAGTCAAC-3′ |
| GAPDH reverse: 5′- CGCTCCTGGAAGATGGTGAT-3′ |
| MFSD12 forward: 5′- TCACCTACTCGCTCCACCTG-3′ |
| MFSD12 reverse: 5′- GGCCTGAGAAGTAGGTCATGT-3′ |
| miR-4732-3p forward: 5′- GATGCCCTGACCTGTCCTGT-3′ |
| U6 forward: 5′- ATTGGAACGATACAGAGAAGATT-3′ |
| hnRNPK siRNA#1: 5′- GAGUCUAGCAGGAGGAAUUTT-3′  3′- AAUUCCUCCUGCUAGACUCTT-5′ |
| hnRNPK siRNA#2: 5′- CAGAAUGGCAGAUGGCUUATT-3′  3′- UAAGCCAUCUGCCAUUCUGTT-5′ |
| hnRNPK forward: 5′- GACAGAATGCCTCCTGGTCG-3′ |
| hnRNPK reverse: 5′- GGAAGATTCCGAGCTCTGCT-3′ |
| miR-92b-3p forward: 5′- UCUUUGGUUAUCUAGCUGUAUGA-3′ |
| miR-1180-3p forward: 5′- TTCCGGCTCGCGTGGGTGTGT-3′ |
| hsa-miR-4732-3p mimics: 5′- GCCCUGACCUGUCCUGUUCUG-3′  3′- CAGAACAGGACAGGUCAGGGC-5′ |
| hsa-miR-4732-3p inhibitors: 5′- CAGAACAGGACAGGUCAGGGC-3′ |
| hsa-miR-NC: 5′- UCACAACCUCCUAGAAAGAGUAGA-3′  3′- UCUACUCUUUCUAGGAGGUUGUGA-5′ |
