# Supplementary material for: Tumor-suppressive miR-4732-3p is sorted into fucosylated exosome by hnRNPK to avoid the inhibition of lung cancer progression
Source: J Exp Clin Cancer Res. 2024 Apr 23;43:123. doi: 10.1186/s13046-024-03048-1 (PMC11036635; doi:10.1186/s13046-024-03048-1)
Supplement: Supplementary file 2 — Supplementary Material 2. [file 13046_2024_3048_MOESM2_ESM.zip › Table S2 Clinical characteristics.docx]

**Table S2. Clinical characteristics of NSCLC, BPN, HC group**

| Variable | NSCLC (n = 96) | | | BPN (n = 30) | | | HC (n = 32) | | | P-*value* |
| --- | --- | --- | --- | --- | --- | --- | --- | --- | --- | --- |
|  | No. Mean | | % | No. Mean | | % | No. Mean | | % |  |
| **Age (years)** |  |  | |  |  | |  |  | | 0.6718 |
| Mean | 53.48  6.942 | | | 52.20  7.604 | | | 53.50  7.044 | | |  |
| Standard deviation |  |  |  |  |  |  |  |  |  |  |
| **Gender** |  |  | |  |  | |  |  | | 0.3208 |
| Male | 70 | 72.92 | | 22 | 73.33 | | 19 | 59.38 | |  |
| Female | 26 | 27.08 | | 8 | 26.67 | | 13 | 40.62 | |  |
| **Type** |  |  | |  |  | |  |  | |  |
| LUAD | 51 | 0.53 | |  |  | |  |  | |  |
| LUSC | 43 | 0.45 | |  |  | |  |  | |  |
| Large cell lung cancer | 2 | 0.02 | |  |  | |  |  | |  |
| **Stage** |  |  | |  |  | |  |  | |  |
| 0 | 31 | 0.32 | |  |  | |  |  | |  |
| I/Ⅱ | 34 | 0.35 | |  |  | |  |  | |  |
| Ⅲ/Ⅳ | 31 | 0.32 | |  |  | |  |  | |  |
