# Supplementary material for: Tumor-suppressive miR-4732-3p is sorted into fucosylated exosome by hnRNPK to avoid the inhibition of lung cancer progression
Source: J Exp Clin Cancer Res. 2024 Apr 23;43:123. doi: 10.1186/s13046-024-03048-1 (PMC11036635; doi:10.1186/s13046-024-03048-1)
Supplement: Supplementary file 2 — Supplementary Material 2. [file 13046_2024_3048_MOESM2_ESM.zip › Table S3 Data of antibodies.docx]

**Table S3. Data of antibodies**

| **Antibody** | **Catalog number** | **Company** | **MW(kDa)** |
| --- | --- | --- | --- |
| CD9 | ET1601-9 | HUABIO | 20 |
| TSG101 | ab125011 | Abcam | 44 |
| Calnexin | ET1611-86 | HUABIO | 100 |
| hnRNPK | ab52600 | Abcam | 60 |
| MFSD12 | orb1553832 | [Biorbyt](http://biorbyt.com.cn/) | 60 |
| p-AKT | 4060 | Cell Signaling Technology | 56 |
| AKT | 4691 | Cell Signaling Technology | 56 |
| P53 | 2524 | Cell Signaling Technology | 53 |
| P21 | ab109199 | Abcam | 21 |
| CDC25C | ab32444 | Abcam | 60 |
| CDK1 | ab133327 | Abcam | 34 |
| CyclinB1 | ab32053 | Abcam | 55 |
| Ki-67 | 12202S | Cell SignalingTechnology |  |
| GAPDH | 5174 | Cell Signaling Technology | 36 |
| β-Tubulin | 2128 | Cell Signaling Technology | 55 |
